# Quercetin alleviates LPS/iE-DAP-induced liver injury by suppressing ferroptosis via regulating ferritinophagy and intracellular iron efflux

**DOI:** 10.1016/j.redox.2025.103557

**Published:** 2025-02-18

**Authors:** Hongzhu Zhang, Huimin Shi, Xuerui Li, Shendong Zhou, Xiaokun Song, Nana Ma, Meijuan Meng, Guangjun Chang, Xiangzhen Shen

**Affiliations:** Ministry of Education Joint International Research Laboratory of Animal Health and Safety, College of Veterinary Medicine, Nanjing Agricultural University, Nanjing, 210095, Jiangsu, PR China

**Keywords:** Ferroptosis, Ferritinophagy, Iron metabolism, Ruminal dysbiosis, Liver injury

## Abstract

Ruminal dysbiosis-induced liver injury is prevalent in dairy cows, yet its underlying mechanisms remain incompletely understood. Ferroptosis, a newly identified form of programmed cell death distinct from apoptosis and necrosis, has been implicated in various liver diseases by emerging studies. In the present study, lipopolysaccharide (LPS) and γ-D-glutamyl‐meso‐diaminopimelic acid (iE-DAP) were employed to establish *in vitro* and *in vivo* models of liver injury using bovine hepatocytes and mice, respectively. It was observed that noncytotoxic iE-DAP alone did not influence lipid peroxidation or GPX4, but exacerbated LPS-induced ferroptosis and hepatocyte injury. Notably, co-treatment with LPS and iE-DAP (LPS/iE-DAP)-induced hepatocyte injury was mitigated by intervention with the ferroptosis inhibitor ferrostatin-1 (Fer-1). Mechanistically, the activated IL-6/STAT3 signaling pathway was found to mediate LPS/iE-DAP-induced ferroptosis. Suppression of IL-6/STAT3, either through *IL6* and *STAT3* knockdown or pharmacological intervention, reduced Fe^2+^ accumulation and alleviated ferroptotic cell death. Further investigations identified that IL-6/STAT3 signaling enhanced ferritinophagy and impaired iron export. Either disrupting ferritinophagy by knocking down *NCOA4* or restoring iron export via *HAMP* knockdown relieved intracellular iron overload and inhibited ferroptosis. Specifically, LPS/iE-DAP treatment increased the interaction between hepcidin and ferroportin, promoting ferroportin ubiquitination and degradation, thereby blocking iron efflux. Furthermore, we provided several evidence to prove that quercetin pretreatment alleviated LPS/iE-DAP-induced ferroptosis and liver injury by decreasing hepatic iron accumulation via targeting the IL-6/STAT3 signaling *in vitro* and *in vivo*, effects reversed by the addition of recombinant bovine IL-6. Based on these findings, we concluded that LPS/iE-DAP-induced liver injury by triggering ferroptosis through regulating IL-6/STAT3/ferritinophagy-dependent iron release and IL-6/STAT3/hepcidin/ferroportin-dependent iron export, while quercetin could alleviate this liver injury by inhibiting ferroptosis via IL-6/STAT3 signaling pathway. This study provides novel insights into the mechanisms whereby ruminal dysbiosis induces liver injury and presents a prospective solution for ruminal dysbiosis-induced liver injury.

## Graphical abstract

**Figure:**
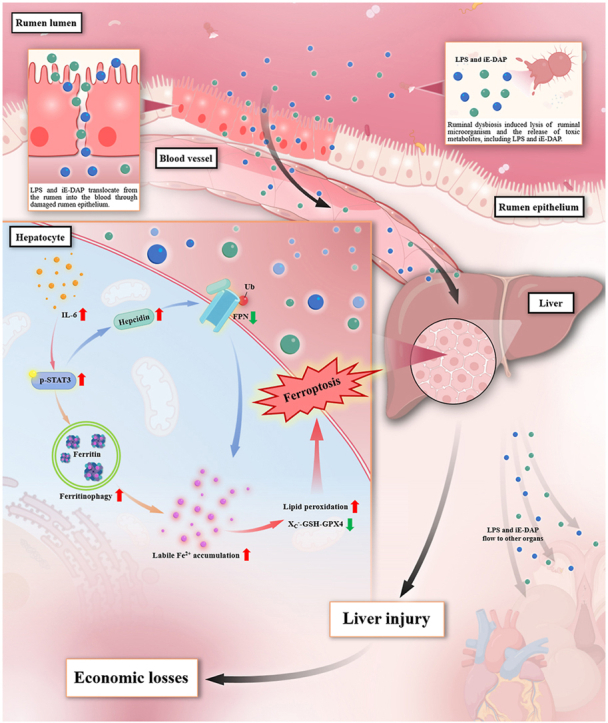

## 1 Introduction

Liver injury is a common disease in dairy cows with multifactorial causes. In addition to fatty liver, ruminal dysbiosis has been identified as a significant contributor to liver injury [1,2]. Feeding high-grain diets is an effective strategy to increase milk production, but often leads to ruminal dysbiosis characterized by reduced ruminal pH, damage to the rumen epithelium, and the lysis of ruminal microorganisms. Pathogenic metabolites derived from lysed rumen bacteria, including lipopolysaccharide (LPS), translocate from the rumen into the bloodstream and subsequently reach the liver, imposing a substantial burden and potentially causing liver injury [3]. Many studies have reached a consensus that rumen-derived LPS is one of the factors contributing to liver injury. Sustained exposure to LPS impairs hepatic function and induces inflammatory responses [4,5]. Our previously published study also confirmed liver injury and increased LPS content in the portal vein of animals with ruminal dysbiosis [6]. In addition to LPS, γ-D-glutamyl‐meso‐diaminopimelic acid (iE-DAP) is another pathogenic metabolite in the rumen. iE-DAP is a dipeptide and is located within the peptidoglycan of gram-negative bacteria and most gram-positive bacteria [7]. It can be recognized by the nucleotide oligomerization domain binding protein1 (NOD1), a specific cytoplasmic receptor, therefore activating downstream nuclear factor-kappa B (NF-κB) and promoting the transcription of inflammatory cytokines [8]. Our previous study confirmed that iE-DAP induces inflammatory damage to bovine mammary epithelial cells [9]. Extensive research has focused on the role of LPS in ruminal dysbiosis-induced liver injury, while the contribution of iE-DAP to this pathological process has been largely overlooked.

The term ferroptosis was first coined by Stockwell et al., in 2012 [10] and has attracted increasing attention in recent years. Ferroptosis, an iron-dependent form of programmed cell death, is distinct from apoptosis, necrosis and autophagy in morphology, biochemistry and genetics [10]. An overload of intracellular iron facilitates lipid peroxidation, which damages cell membranes and causes cells to shrink into a balloon-like shape, a characteristic phenotype of ferroptosis [11]. There is a functional system within cells to counteract ferroptosis. The glutathione peroxidase-4 (GPX4)-mediated cellular defense system is considered a critical surveillance mechanism against ferroptosis, as GPX4 can catalyze the reduction of toxic lipid peroxides to nontoxic products [12]. Upregulating GPX4 inhibits ferroptosis [13], whereas GPX4 degradation drives ferroptosis [14,15]. As the term itself implies, ferroptosis is intricately regulated by iron metabolism. Ferritin, composed of ferritin heavy chain (FTH) and ferritin light chain (FTL), functions as a reservoir for excessive intracellular iron [16]. As a result, ferritin metabolism is intimately implicated in the initiation of ferroptosis. Ferritin could bind to the nuclear receptor coactivator (NCOA4) and be transported to lysosomes for degradation, a process defined as ferritinophagy, causing the release of labile iron [17]. In addition, intracellular iron homeostasis also relies on the balance between iron absorption and iron export [18]. To date, the relationship between ferroptosis and iron metabolism in ruminal dysbiosis-induced liver injury has not been explored.

Quercetin is a flavonoid abundant in fruits and vegetables [19]. In recent years, quercetin has garnered significant attention due to its anti-inflammatory, anti-oxidant, anti-viral, and anti-carcinogenic properties [20]. Studies have demonstrated that quercetin could alleviate fatty liver by ameliorating inflammation and oxidative stress [21]. Furthermore, recent findings suggest that quercetin mitigates deoxynivalenol-induced intestinal injury [22] and acrylamide-induced liver injury [23] by inhibiting ferroptosis, indicating ferroptosis as a potential target of quercetin. However, whether quercetin could be used to treat ruminal dysbiosis-induced liver injury needs to be investigated.

In the present study, we aimed to investigate the liver injury triggered by LPS and iE-DAP co-treatment. To this end, bovine hepatocytes were treated with LPS and iE-DAP to imitate liver injury in dairy cows caused by rumen-derived pathogenic metabolites, and ferroptosis and iron metabolism were investigated. Next, hepatocytes were pretreated with quercetin to evaluate the effects of quercetin on LPS/iE-DAP-induced ferroptosis and hepatocyte injury, as well as to explore the underlying mechanisms. Finally, a liver injury model was established in mice, and quercetin was administered orally to further validate the *in vitro* findings. This study provides new insights into the mechanisms of ruminal dysbiosis-derived liver injury in cows and offers a prospective approach to mitigate such disease.

## 2 Materials and methods

### 2.1 Reagents

Lipopolysaccharide (Cat #L2630) was purchased from Sigma-Aldrich (St Louis, MO, USA). iE-DAP (Cat # tlrl-dap) was obtained from InvivoGen (San Diego, CA, USA). Quercetin (IQ0010), cell counting kit-8 (CCK-8, CA1210), and iron assay kit (BC5415) were purchased from Solabio (Beijing, CHN). Deferoxamine mesylate (DFOM, Cat # HY-B0988), ferrostatin-1 (Fer-1, HY-100579), Stattic (HY-13818), Tocilizumab (HY–P9917), and MG-132 (HY-13259) were supplied by Med Chem Express (Monmouth Junction, NJ, USA). BODIPY 581/591 C11 (D3861) was purchased from Invitrogen (Carlsbad, CA, USA). Rabbit anti-acyl-CoA synthetase long-chain family member 4 (ACSL4, 66617-1-Ig, 1:1000 for western blot), mouse anti-GPX4 (67763-1-Ig, 1:1000 for western blot, 1:200 for immunofluorescence), rabbit anti-solute carrier family 7 member 11 (SLC7A11, 26864-1-AP, 1:1000 for western blot), rabbit anti-solute carrier family 3 member 2 (SLC3A2, 15193-1-AP, 1:1000 for western blot), rabbit anti-phospho-signal transducer and activator of transcription 3 (p-STAT3, 28945-1-AP, 1:1000 for western blot, 1:200 for immunofluorescence), mouse anti-STAT3 (60199-1-Ig, 1:1000 for western blot), rabbit anti-ferritin heavy chain (FTH, 11682-1-AP, 1:1000 for western blot, 1:200 for immunofluorescence), mouse anti-lysosomal associated membrane protein2 (LAMP1, 67300-1-Ig, 1:200 for immunofluorescence), rabbit anti-NCOA4 (83394-4-RR, 1:1000 for western blot, 1:300 for immunofluorescence), rabbit anti-microtubule-associated protein 1 light chain 3 (MAP1LC3, 81004-1-RR, 1:300 for immunofluorescence), rabbit anti-divalent metal transporter 1 (DMT1, 20507-1-AP, 1:1000 for western blot), rabbit anti-ferroportin (FPN, 26601-1-AP, 1:1000 for western blot), rabbit anti-ubiquitin (10201-2-AP, 1:2800 for western blot), mouse anti-glyceraldehyde-3-phosphate dehydrogenase (GAPDH, 60004-1-Ig, 1:1000 for western blot), corallite 488-conjugated goat anti-mouse IgG (H + L) (SA00013-1), and corallite 594-conjugated goat anti-rabbit IgG (H + L) (SA00013-4) were purchased from Proteintech (Rosemont, IL, USA). Rabbit anti-hepcidin (DF6492, 1:1000 for western blot) and rabbit anti-transferrin receptor (TFR, AF5343, 1:1000 for western blot) was purchased form Affinity Biosciences (Jiangsu, CHN). Rabbit anti-arachidonate 15-lipoxygenase (ALOX15, AG1096, 1:1000 for western blot), mouse anti-interleukin 6 (IL-6, AF7236, 1:1000 for western blot), rabbit anti-phospho–NF–κB (AF5881, 1:1000 for western blot), rabbit anti–NF–κB (AF1234, 1:1000 for western blot), rabbit anti-Toll-like Receptor 4 (TLR4, AF8187, 1:1000 for western blot), rabbit anti-phospho-IκB (AF1870, 1:1000 for western blot), rabbit anti-STAT1 (AG3318, 1:1000 for western blot), rabbit anti-phospho-STAT1 (AF2212, 1:1000 for western blot), rabbit anti-STAT5 (AG3325, 1:1000 for western blot), rabbit anti-phospho-STAT5 (AF5947, 1:1000 for western blot), mouse anti-Histone H3 (AF0009, 1:1000 for western blot), HRP-labeled goat anti-rabbit IgG (H + L) (A0208, 1:1000 for western blot), and HRP labeled goat anti-mouse IgG (H + L) (A0216, 1:1000 for western blot) were obtained from Beyotime Biotech (Beijing, CHN).

### 2.2 Hepatocyte isolation and culture

Bovine hepatocytes were isolated according to a nonperfusion technique from our previously published study [24]. Briefly, 1–3 g of liver tissue was obtained from Holstein cows via puncture biopsy. After standard procedures: cleaning, chopping, centrifugation, incubation with collagenase, and filtration, hepatocytes were obtained and cultured in growth medium (Dulbecco's modified Eagle's medium supplemented with 10 % fetal bovine serum, 1 nM glucagon, 10 nM dexamethasone, 10 ng/mL epidermal growth factor, 10 nM insulin, and 1 % penicillin/streptomycin). Hepatocytes were maintained at 37 °C in a humidified 5 % CO_2_ incubator.

### 2.3 Cell experiment design

Hepatocytes were treated with 20 μg/mL LPS and 5 μg/mL iE-DAP for 12 h to induce hepatocyte injury. To discern the involvement of ferroptosis, ferroptosis inhibitor Fer-1 (10 μM) and iron chelator DFOM (5 μM) were added to hepatocytes for further study. To investigate the impact of IL-6/STAT3 signaling on ferritinophagy and ferroptosis, Stattic (5 μM), and Tocilizumab (20 μg/mL) were added to the culture medium 4 h before LPS/iE-DAP treatment. For quercetin and rbIL-6 experiments, quercetin (20 μM) and rbIL-6 (10 ng/mL) were added to culture medium for 24 h. LD refers to LPS and iE-DAP co-treatment.

### 2.4 Cell viability assay

Cell viability was measured with a CCK-8 kit. Briefly, hepatocytes were seeded in a 96-well plate at a density of 1 × 10^4^/well. After 12 h, hepatocytes were administered with corresponding drugs. When drug treatment was finished, the medium was discarded and 110 μL of new medium containing 10 μL CCK8 reagent was added followed by incubation at 37 °C for 2 h. The absorbance value at 450 nm was measured with SPARK® microplate reader (TECAN, Switzerland).

### 2.5 Cell transfection

Small interfering RNA against bovine *IL6*, *STAT3*, *NCOA4*, and *HAMP* were synthesized by Sangon Biotech (Shanghai, CHN). Hepatocytes were seeded into 6-cm plates for protein analysis (6 × 10^5^ cells/plate) or 24-well plates for other analysis (8 × 10^4^ cells/well). The hepatocytes were transfected with siRNA with a Lipofectamine 3000 kit (L3000015, Thermo Fisher Scientific) according to the manufacturer's protocol.

### 2.6 Animals and animal model

A total of ninety C57 mice (male, 8-week-old) were supplied by Anuokang Biotech (Nanjing, CHN). All animal experiments were conducted under the Guidelines for the Care and Use of Laboratory Animals of Nanjing Agricultural University. Animals were housed in a standard condition with room temperature of 22–24 °C and 12 h light/dark cycle and had free access to food and water. All mice underwent one week of acclimation before the formal experiment.

The concentration of iE-DAP applied in our experiments referred to the study published by Carmen et al. [25]. To determine the optimal concentration of iE-DAP, mice were randomly divided into five groups: Control group, LPS group (injected intraperitoneally with 10 mg/kg LPS), and three LPS + iE-DAP groups with different iE-DAP concentrations (LDAP1 group, 10 mg/kg LPS + 1 mg/kg iE-DAP; LDAP2, 10 mg/kg LPS + 2 mg/kg iE-DAP; LDAP3, 10 mg/kg LPS + 3 mg/kg iE-DAP) (n = 6 per group).

For the Fer-1 intervention experiment, mice were randomly divided into four groups: Control group, LD group, Fer-1 group, and LD + Fer-1 group (n = 6 per group). In this experiment, mice were intraperitoneally injected with 1 mg/kg Fer-1 or equal volume of normal saline daily for 3 d before LPS/iE-DAP injection.

For the quercetin intervention experiment, mice were randomly divided into six groups: Control group, LD group, Stattic group, quercetin group, LD + quercetin group, and LD + Stattic group (n = 6 per group). For the LD group, mice were gavaged with normal saline for 21 d, and injected intraperitoneally with 10 mg/kg LPS and 3 mg/kg iE-DAP on d 22; for the LD + quercetin group, mice were gavaged with 50 mg/kg quercetin daily for 21 d before LPS/iE-DAP injection; for LD + Stattic treatment, mice were injected intraperitoneally with 10 mg/kg Stattic daily for 7 d before LPS/iE-DAP injection. All mice were anesthetized and sacrificed by cervical decapitation 12 h after LPS or iE-DAP injection. Blood and liver tissue were collected for subsequent research.

### 2.7 RNA isolation and transcriptome analysis

The total RNA of hepatocytes and liver tissues was extracted with a FastPure Cell/Tissue Total RNA Isolation Kit V2 (RC112, Vazyme, Nanjing, CHN). The RNA integrity and concentration were measured using the Qubit 400 bioanalyzer. Library sequencing was performed with a HiSeq X system (Illumina) and library quality was confirmed using Agilent 2100. Stats 3.5.1 package (R 3.5.1) was used to conduct principal component analysis (PCA). Ggplot2 3.3.0 package (R 3.5.1) was used to performed differential expression analysis, and the threshold of *P*-value <0.05 and Fold Change >2 were set for significantly differential expression. KEGG pathway enrichment analysis was performed on the differentially expressed genes with a ggplot2 3.3.0 package (R 3.5.1).

### 2.8 Real-time quantitative PCR (RT-qPCR)

After RNA isolation, cDNA was obtained by using a HiScript Ⅲ RT SuperMix for Qpcr (R323, Vazyme). RT-qPCR assay was conducted using a ChamQ Universal SYBR qPCR Master Mix (Q711, Vazyme) according to the manufacturer's protocol. The primers used in this experiment were designed by Oligo 7 software and listed in the Supplementary Table 1. *GAPDH* was used as an internal reference, and the 2^−ΔΔCt^ method was used for analysis.

### 2.9 Western blot and co-immunoprecipitation assays

Liver tissues or hepatocytes were lysed with RIPA lysis buffer purchased from YEASEN (20101 ES, Shanghai, CHN), and protein concentrations were measured with a BCA assay kit (ZJ101, Epizyme Biotech, Shanghai, CHN). Proteins (20 μg) of each sample were separated in a 10 % SDS-PAGE gel and transferred to the PVDF membrane, which was followed by incubation in skim milk at room temperature (RT) for 2 h. Then, membranes were incubated with primary antibody at 4 °C for 12 h, followed by incubation with secondary antibody at RT for 1 h. The membranes were incubated with a chemiluminescence reagent (33222ES76, YEASEN), and brands were detected by a ChemDoc CRS + Imaging System (Bio-Rad, Hercules, USA). Image J software was used to quantify the brands. The primary antibodies and secondary antibodies were listed below: ACSL4, ALOX15, GPX4, SLC7A11, SLC3A2, FTH, IL-6, p-STAT1, STAT1, p-STAT3, STAT3, p-STAT5, STAT5, TFR, DMT1, Hepcidin, FPN, NCOA4, GAPDH, HRP-labeled goat anti-rabbit IgG (H + L), and HRP labeled goat anti-mouse IgG (H + L).

For the co-immunoprecipitation (co-IP) assay, hepatocytes were washed with PBS and lysed on ice using an IP lysis buffer added with protease inhibitor cocktail (K1007, APEXBIO, USA). Lysates were incubated with anti-MYC for immunoprecipitation. For each IP reaction, antibody (2–4 μg) was combined with 1 mL of cell lysate. Following overnight incubation at 4 °C, protein A + G agarose beads (P2012, Beyotime) were added, and after 1 h, the beads were rinsed. Immunoprecipitates were subsequently eluted with SDS loading buffer. The supernatants were analyzed by western blot with specific antibodies.

### 2.10 Indirect immunofluorescence analysis

Hepatocytes were cultured in a 24-well plate with coverslips (8 × 10^4^ cells/well) and fixed with 4 % paraformaldehyde when the drug treatment was finished. After permeabilization in 0.2 % Triton X-100 for 10 min and incubation in 1 % BSA for 30 min, each well was added 250 μL primary antibody and incubated at 4 °C for 12 h. Then, cells were incubated with secondary antibody at RT for 1 h, followed by DAPI staining for 10 min. Fluorescence was detected with a confocal microscope (LSM710, Carl Zeiss, Jena, Germany).

### 2.11 Measurement of Fe^2+^ content

Cellular and hepatic Fe^2+^ contents were measured with an iron detection kit (BC5415) from Solarbio. All procedures were performed according to the manufacturer's protocol. The absorbance value at 593 nm was measured with SPARK® microplate reader (TECAN) and the iron content of each sample was obtained according to the standard curve.

### 2.12 Lipid ROS detection

Hepatocytes were cultured in a 24-well plate (8 × 10^4^ cells/well) and incubated with 300 μL DMEM containing 5 μM BODIPY 581/591 C11 at 37 °C for 30 min. Then, cells were washed with PBS. Fluorescence was detected with a confocal microscope (LSM710, Carl Zeiss).

For flow cytometry, hepatocytes were seeded in 12-well plates (1.6 × 10^5^ cells/well). After incubation with DMEM containing BODIPY 581/591 C11, hepatocytes were collected in 1.5-mL tube and centrifuged 2 times followed by PBS resuspension. 10,000 cells of each sample were collected and the proportion of lipid ROS-positive cells was analyzed with a flow cytometer (Accuri C6, BD Biosciences, San Jose, CA, USA).

### 2.13 Malondialdehyde (MDA) measurement

MDA content was measured with an MDA assay kit (A003-1-2, Jiancheng Bioengineering, Nanjing, CHN). The absorbance value at 532 nm was measured with SPARK® microplate reader (TECAN). MDA content was calculated according to the manuscript's protocol.

### 2.14 Glutathione (GSH) measurement

Reduced GSH, total GSH, and oxidized glutathione disulfide (GSSG) were accessed with a GSH assay kit (061-1-1) from Jiancheng Bioengineering. The absorbance value at 405 nm was measured with SPARK® microplate reader (TECAN). The contents of total GSH and GSSG were calculated based on the standard curve. Reduced GSH content was measured based on the below formula: C_reduced-GSH_ = C_total-GSH_-C_GSSG_ × 2.

### 2.15 IL-6 measurement

Serum IL-6 was accessed with an enzyme-linked immunosorbent assay (ELISA) kit (H007-1-1, Jiancheng Bioengineering) according to the manufacturer's protocol. The absorbance value at 450 nm was measured with SPARK® microplate reader (TECAN). IL-6 content was calculated based on the standard curve.

### 2.16 Immunohistochemistry

Liver tissues in 4 % paraformaldehyde were embedded in paraffin and cut into sections with a thickness of 4 μm. After the slices were dewaxed, antigens were retrieved using sodium citrate solution. The slices were incubated with corresponding primary antibody, secondary antibody, and DAPI. Images were captured using a microscope (EVOS FL AUTO, Invitrogen).

### 2.17 Hematoxylin-eosin (H&E) staining

Liver tissues in 4% paraformaldehyde were embedded in paraffin and cut into sections with a thickness of 4 μm. After dewaxing, the slices were stained with hematoxylin and eosin dyes. The histopathological changes were observed with a microscope (EVOS FL AUTO, Invitrogen).

### 2.18 Statistical analysis

All statistical analyses were performed using SPSS 26.0 (IBM Inc., Armonk, NY). Data were tested for normality of distribution with the Shapiro-Wilk test. Data between the two groups were analyzed using an independent-sample *t*-test. Other data were analyzed using one-way ANOVA with Dunnett's post-testing. The results are expressed as the mean and standard error of the mean (mean ± SEM). For all analyses, *P*-values <0.05 were considered statistically significant.

## 3 Results

### 3.1 Cows with ruminal dysbiosis developed liver injury and increased level of hepatic ferroptosis

To investigate the association between ruminal dysbiosis-derived liver injury and hepatic ferroptosis, we collected liver and blood samples from 12 cows, six of which were healthy and six of which were with ruminal dysbiosis (RD). Liver injury in the RD group was confirmed through the analysis of serum alanine aminotransferase (ALT) and aspartate aminotransferase (AST) levels, which exceeded the normal ranges and were significantly higher than those in the healthy group (Fig. S1A). Ruminal dysbiosis led to hepatic Fe^2+^ accumulation, elevated MDA formation (a lipid peroxidation product), and reduced serum GPX4 level (Figs. S1B–D). Western blot analysis further revealed differences in lipid peroxidation-related proteins (ACSL4 and ALOX15) and system x_c_^−^-GPX4 pathway proteins (GPX4, SLC3A2, and SLC7A11) between healthy cows and cows with ruminal dysbiosis. Specifically, cows with ruminal dysbiosis exhibited increased protein expression of ACSL4 and ALOX15 and decreased protein expression of GPX4, SLC3A2, and SLC7A11 (Figs. S1E–F), indicative of enhanced lipid peroxidation and reduced anti-lipid peroxidation capacity in the livers. The data above implied that ruminal dysbiosis-derived liver injury may be related to an increased level of hepatic ferroptosis.

### 3.2 iE-DAP aggravated LPS-induced hepatocytes injury and ferroptosis

To explore the mechanisms of RD-induced liver injury, LPS and iE-DAP, two known rumen-derived pathogenic metabolites, were selected to establish an *in vitro* liver injury model. To evaluate the effects of iE-DAP and LPS on bovine hepatocytes, a cell viability assay was performed. As shown in F ig. S2A, iE-DAP significantly reduced cell viability at a concentration of 10 μg/mL and induced damage to cells in a dose-dependent manner. Given that iE-DAP is not the primary pathogenic molecule in ruminal dysbiosis-derived liver injury, the effects of noncytotoxic concentrations of iE-DAP on ferroptosis were examined. Cellular Fe^2+^ content was significantly elevated at a dose of 3 μg/mL iE-DAP and peaked at 5 μg/mL iE-DAP (Fig. S2B). However, no significant differences in MDA content, gene expression of *ACSL4* and *GPX4*, and protein expression of ACSL4 and GPX4 were observed among the groups (Figs. S2C–F), indicating that noncytotoxic iE-DAP did not influence ferroptosis. Based on these findings, 5 μg/mL iE-DAP was selected for subsequent experiments.

**Fig. 1:**
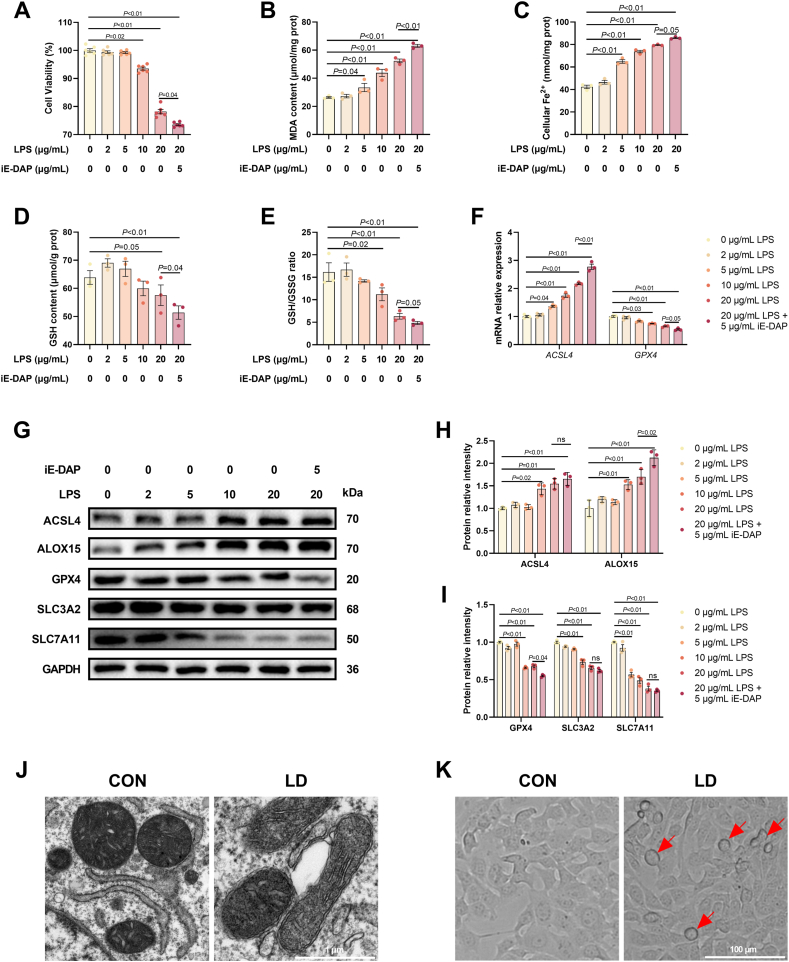
**iE-DAP aggravated LPS-induced ferroptosis in hepatocytes.** (A) Effect of different concentrations of iE-DAP on cell viability; n = 6 per group. (B) MDA content, (C) cellular Fe^2+^, (D) GSH content, and (E) GSH/GSSG ratio in hepatocytes treated with different concentrations of LPS or the combination of LPS and iE-DAP; n = 3 per group. (F) The mRNA expression of *ACSL4* and *GPX4* and (G–I) protein expression of ACSL4, ALOX15, GPX4, SLC7A11 and SLC3A2 in hepatocytes with different treatments; n = 3 per group. (J) Mitochondria images in hepatocytes captured by transmission electron microscopy, scale bar = 1 μm. (K) Hepatocyte morphology captured at phase difference field and red arrow indicates ballooning cells, scale bar = 100 μm. Data are presented as mean ± SEM.

Different doses of LPS were applied to hepatocytes, and the results showed that 10 and 20 μg/mL LPS significantly reduced cell viability. Additionally, the combination of 5 μg/mL iE-DAP and 20 μg/mL LPS (referred henceforth simply as LPS/iE-DAP) further decreased cell viability (Fig. 1A). As shown in Fig. 1B–E, LPS treatment increased MDA content and cellular Fe^2+^, and decreased GSH (an antioxidant enzyme) content and GSH/GSSG ratio in a dose-dependent manner. LPS/iE-DAP further amplified these effects, showing higher cellular Fe^2+^ and more MDA formation as well as lower GSH content and GSH/GSSG ratio compared to the 20 μg/mL LPS group. Moreover, LPS treatment dose-dependently upregulated the gene expression of *ACSL4* and the protein expression of ACSL4 and ALOX15, and dose-dependently decreased the gene expression of *GPX4* and the protein expression of GPX4, SLC3A2 and SLC7A11 (Fig. 1F–I). Notably, these changes in the gene expression of *ACSL4* and *GPX4* as well as the protein expression of ALOX15 and GPX4 caused by LPS treatment were exacerbated by LPS/iE-DAP treatment (Fig. 1F–I). In addition, LPS/iE-DAP caused mitochondrial damage characterized by ruptured membranes and reduced cristae (Fig. 1J), along with an increased number of balloon-like cells (typical morphology of ferroptotic cells) (Fig. 1K). Therefore, we concluded that noncytotoxic iE-DAP could facilitate LPS-induced hepatocyte injury; moreover, the increased cellular Fe^2+^, enhanced lipid peroxidation, and impaired system x_c_^−^-GPX4 caused ferroptosis. A combination of 20 μg/mL LPS and 5 μg/mL iE-DAP was selected to establish a hepatocyte injury model for subsequent studies.

### 3.3 Inhibition of ferroptosis alleviated LPS/iE-DAP-induced hepatocyte injury

**Fig. 2:**
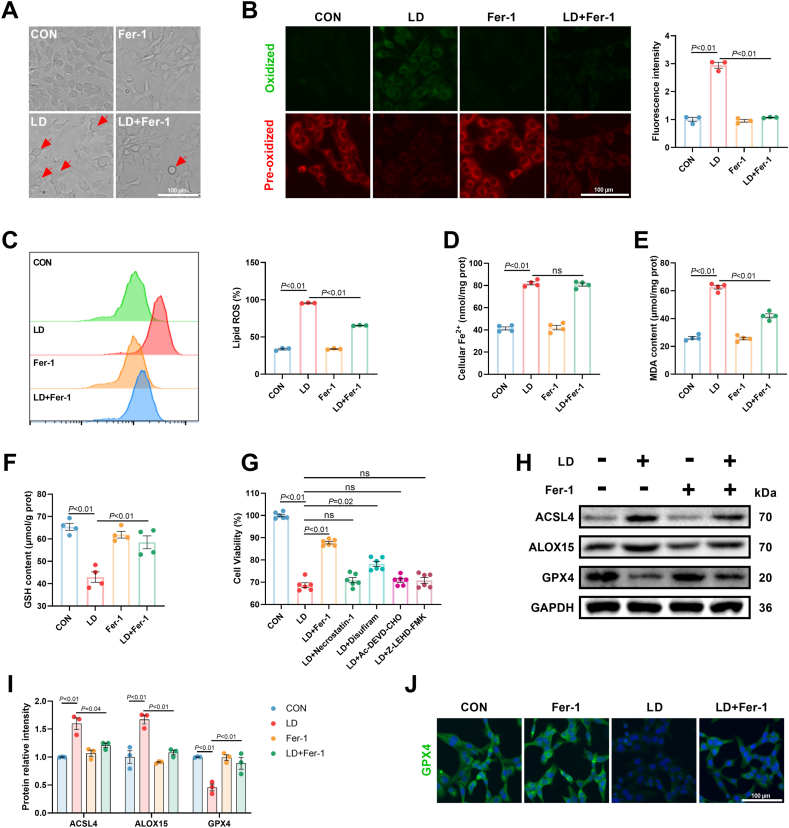
**Fer-1 intervention inhibited ferroptosis and relieved cell injury in hepatocytes treated with LPS/iE-DAP.** (A) Cell morphology was observed through phase-contrast pattern of optical microscope and red arrow indicates ballooning cells, scale bar = 100 μm. (B–C) Fluorescence images of lipid ROS observed by confocal microscope (Scale bar = 100 μm) and proportion of lipid ROS positive cells analyzed by flow cytometer; n = 3 per group. (D) cellular Fe^2+^, (E) MDA content, and (F) GSH content in hepatocytes of different groups; n = 4 per group. (G) Cell viability of hepatocytes treated with Fer-1, Necrostatin-1, Disufiram, Ac-DEVD-CHO, and Z-LEHD-FMK was measured with a CCK-8 kit; n = 6 per group. (H–I) protein expression of ACSL4, ALOX15, and GPX4 in hepatocytes with different treatments; n = 3 per group. (J) Immunofluorescence analysis of GPX4, scale bar = 100 μm. Data are presented as mean ± SEM.

To examine the possibility that the observed increased level of ferroptosis was a cause of LPS/iE-DAP-induced hepatocyte injury, ferrostatin-1 (Fer-1), a ferroptosis antagonist, was employed. Fer-1 effectively alleviated ferroptotic cell death, as evidenced by a decreased number of balloon-like cells (Fig. 2A). Lipid ROS level was significantly decreased in Fer-1-treated cells, as verified through both fluorescence observation and flow cytometry (Fig. 2B and C). Furthermore, Fer-1 intervention reversed LPS/iE-DAP-induced reductions in cell viability and GSH content, as well as increases in MDA formation; however, cellular Fe^2+^ content remained unaffected (Fig. 2D–G). Intriguingly, LPS/iE-DAP-induced decreased cell viability was also mitigated by Disufiram (a pyroptosis inhibitor), albeit to a less extent than Fer-1, whereas Necrostatin-1 (a necroptosis inhibitor), Ac-DEVD-CHO (a caspase-3 inhibitor to inhibit apoptosis), and Z-LEHD-FMK (a caspase-9 inhibitor to inhibit apoptosis) showed no significant effects (Fig. 2G); Among these treatments, Fer-1 exhibited the most pronounced protective effect, indicating the critical role of ferroptosis in LPS/iE-DAP-induced hepatocytes injury. This suggested that among these several types of cell death, ferroptosis contributed the most to LPS/iE-DAP-induced hepatocytes injury. The inhibitory effect of Fer-1 on lipid peroxidation was also confirmed by Western blot analysis, as evidenced by decreased protein expression of ACSL4 and ALOX15 and upregulated GPX4 protein level in the LD + Fer-1 group compared to the LD group (Fig. 2H and I). Consistently, immunofluorescence analysis showed that LPS/iE-DAP treatment decreased GPX4 content, which was restored by Fer-1 (Fig. 2J). Collectively, the results above demonstrated that ferroptosis mediated LPS/iE-DAP-induced hepatocyte injury.

To further verify the role of ferroptosis in LPS/iE-DAP-induced liver injury, an *in vivo* model of LPS/iE-DAP-induced liver injury was established in mice. The doses of LPS and iE-DAP referred to previously published studies [25,26]. Our results showed that LPS injection led to increases in serum ALT and AST, and hepatic Fe^2+^ content, while these increases were further augmented by iE-DAP injection in a dose-dependent manner (Figs. S3A–C). Likewise, LPS/iE-DAP co-injection further increased the protein expression of ACSL4 and ALOX15, and further decreased the protein expression of SLC3A2, SLC7A11, and GPX4 compared to LPS injection alone (Figs. S3D–G). Notably, the most significant changes in all of the above indices were observed at 10 mg/kg LPS and 3 mg/kg iE-DAP, thus these LPS and iE-DAP concentrations were selected for the *in vivo* liver injury model. We found that Fer-1 intervention alleviated LPS/iE-DAP-induced liver injury and hepatic ferroptosis with signs of relieved inflammatory cytokines infiltration, decreased serum ALT and AST levels, and alterations in ferroptosis-related markers, including decreased MDA formation, increased GSH content and GSH/GSSG ratio, decreased protein expression of ACSL4 and ALOX15, and increased protein expression of GPX4 in the liver (Fig. S4). The findings of this *in vivo* study further confirmed the vital role of ferroptosis in LPS/iE-DAP-induced liver injury.

### 3.4 Suppressing IL-6/STAT3 counteracted LPS/iE-DAP-induced ferroptotic cell death

**Fig. 3:**
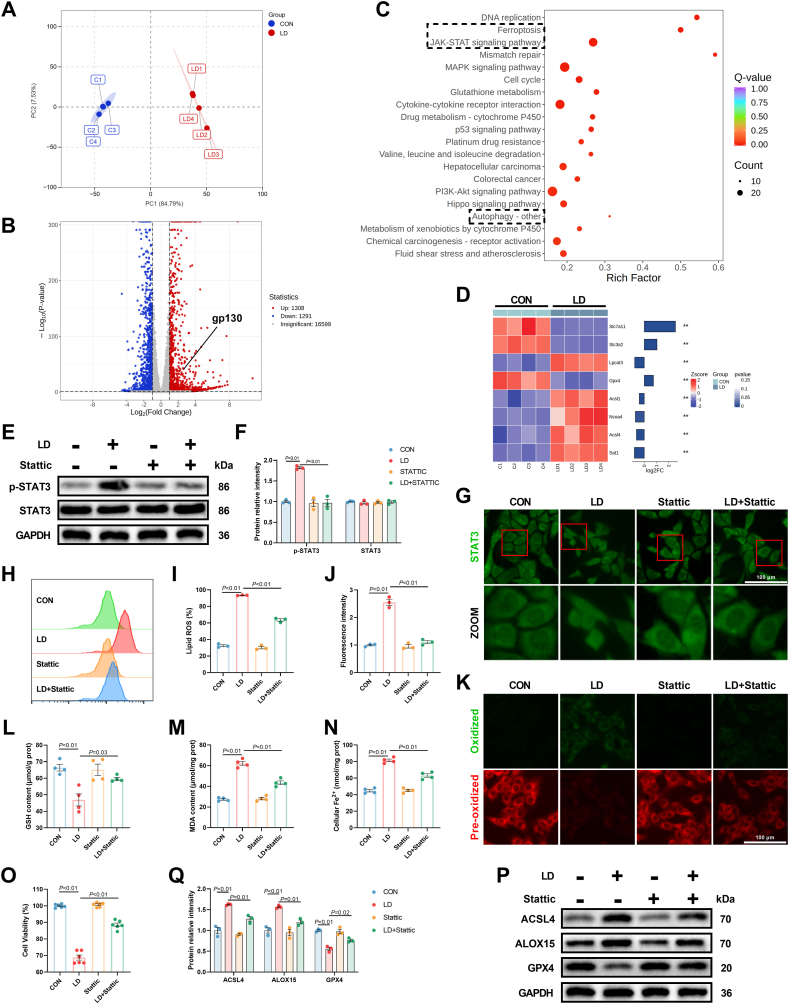
**Stattic intervention suppressed LPS/iE-DAP-induced ferroptotic hepatocytes death.** (A) PCA of transcriptome analysis of hepatocytes in indicated groups. (B) The total differentially expressed genes in indicated groups. (C) The upregulated signaling pathway revealed by KEGG enrichment analysis. (D) Heat map showed representative ferroptosis-related genes expression profiles. (E–F) Protein expression of p-STAT3 and STAT3 in indicated groups; n = 3 per group. (G) Cellular location of STAT3 was detected with immunofluorescence analysis, scale bar = 100 μm. (H–K) Fluorescence images of lipid ROS observed by confocal microscope (Scale bar = 100 μm) and proportion of lipid ROS positive cells analyzed by flow cytometer; n = 3 per group. (L) GSH content, (M) MDA content, (N) cellular Fe^2+^, and (O) cell viability in hepatocytes of different groups; n = 4–6 per group. (P–Q) Protein expression of ACSL4, ALOX15, and GPX4 in indicated groups; n = 3 per group. Data are presented as mean ± SEM.

To systematically explore the mechanisms underlying LPS/iE-DAP-induced hepatocyte injury, a transcriptomic assay was performed on hepatocytes treated with/without LPS/iE-DAP. The within-group repeatability of each group was confirmed through principal component analysis (Fig. 3A), and differentially expressed genes were identified (Fig. 3B). KEGG enrichment analysis revealed notable upregulation of ferroptosis signaling pathways (Fig. 3C). A heat map also showed several significant changed ferroptosis-related genes between groups (Fig. 3D). Additionally, KEGG enrichment analysis displayed that JAK-STAT signaling pathway was upregulated (Fig. 3C). Previous studies have implicated the JAK-STAT signaling pathway in ferroptosis [27]. STATs family contains several members, to identify which STAT family member was involved in LPS/iE-DAP-induced ferroptosis, we measured the changes in STAT1, STAT3, and STAT5, the three major STAT family members often reported in LPS-induced inflammatory responses. Our results indicated that, under LPS/iE-DAP treatment, the phosphorylation levels of all three STATs were significantly increased, while their total protein levels remained unchanged (Figs. S5A–B), suggesting that all three STATs were activated. However, only knockdown of *STAT3* was able to reverse the decreased cell viability caused by LPS/iE-DAP, while knockdown of *STAT1* or *STAT5* had no effect (Fig. S5C). Furthermore, compared to the si-NC + LD group, *STAT3* knockdown decreased cellular Fe^2+^, MDA formation, and protein expression of ACSL4 and ALOX15, while increasing GSH content and protein expression of GPX4 (Figs. S5D–H). We also employed Stattic, an STAT3 antagonist, to further verify the role of STAT3 in ferroptosis. Stattic treatment suppressed LPS/iE-DAP-induced phosphorylation of STAT3 (Fig. 3E and F). Immunofluorescence assay confirmed the inactivation of STAT3, as evidenced by decreased translocation of STAT3 from the cytoplasm into the nucleus (Fig. 3G). Next, we examined ferroptosis-related indicators, and similar to the *STAT3* knockdown experiments, Stattic significantly alleviated ferroptotic cell death with signs of decreased lipid ROS, MDA formation and cellular Fe^2+^, increased GSH content, improved cell viability, decreased protein expression of ACSL4 and ALOX15, and increased protein expression of GPX4 (Fig. 3H–Q). Taken together, these results demonstrated that activated STAT3 mediated ferroptotic cell death in LPS/iE-DAP-treated hepatocytes.

**Fig. 4:**
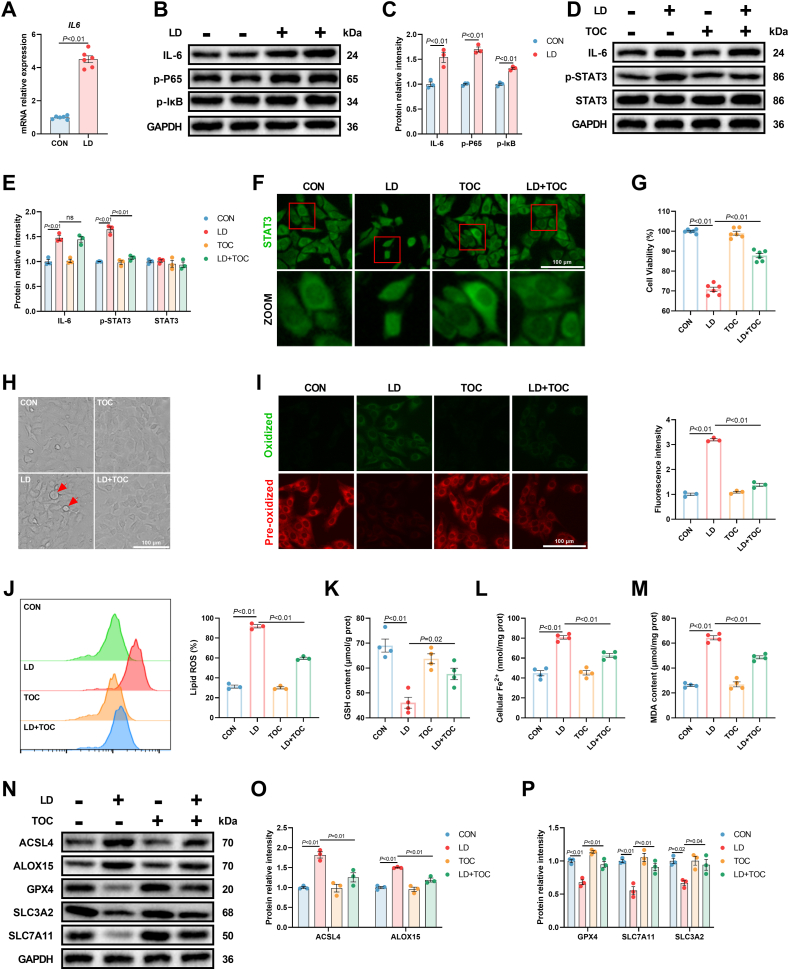
**Inhibition of IL-6 suppressed ferroptosis in hepatocytes treated with LPS/iE-DAP.** (A–C) Gene expression of *IL6* and protein expression of IL-6, p-P65, p-IκB in hepatocytes with or without LPS/iE-DAP; n = 3–6 per group. (D–E) Protein expression of IL-6, p-STAT3, and STAT3 in indicated groups; n = 3 group. (F) Cellular location of STAT3 was detected with immunofluorescence analysis, scale bar = 100 μm. (G) Cell viability was measured with a CCK-8 kit; n = 6 per group. (H) Cell morphology was observed through phase-contrast pattern of optical microscope and red arrow indicates ballooning cells, scale bar = 100 μm. (I–J) Fluorescence images of lipid ROS observed by confocal microscope (Scale bar = 100 μm) and proportion of lipid ROS positive cells analyzed by flow cytometer; n = 3 per group. (K) GSH content, (L) cellular Fe^2+^, and (M) MDA content in different groups; n = 4 per group. (N–P) Protein expression of ACSL4, ALOX15, GPX4, SLC3A2, and SLC7A11 in indicated groups; n = 3 per group. Data are presented as mean ± SEM.

It was well established that gp130 family cytokines are the primary activator of STAT3, and transcriptomic analysis revealed that gp130 was significantly upregulated in hepatocytes treated with LPS/iE-DAP (Fig. 3B). As the primary ligand of gp130, IL-6 was reported to be a STAT3 activator [28]. Our previous studies demonstrated that both LPS and iE-DAP could elevate IL-6 expression [29,30]. The present study confirmed that LPS/iE-DAP treatment increased both gene and protein expression of IL-6 (Fig. 4A–C). Additionally, cows with ruminal dysbiosis showed elevated protein expression of IL-6 and p-STAT3 in the liver, as well as increased serum IL-6 level (Figs. S1G–I). The NF-κB signaling pathway, which is responsible for the transcriptional activation of cytokines such as IL-6, was activated in cows with ruminal dysbiosis, as evidenced by increased nuclear P65 and p-IκB levels (Figs. S1J–K). *In vitro* study also showed that LPS/iE-DAP activated the NF-κB signaling pathway, indicated by elevated p-P65 and p-IκB levels (Fig. 4B and C). Therefore, we hypothesized that increased IL-6 might activate STAT3 and thus mediate LPS/iE-DAP-induced ferroptosis. To this end, Tocilizumab (TOC, inhibiting the binding of IL-6 to IL-6 receptor to suppress the activation of downstream signaling) was employed. We found that Tocilizumab treatment did not significantly change the protein expression of IL-6, but decreased p-STAT3 level and promoted STAT3 translocation from the nucleus to the cytoplasm (Fig. 4D–F), suggesting that IL-6 is essential for STAT3 activation in LPS/iE-DAP-treated hepatocytes. Meanwhile, decreased lipid ROS, MDA formation, and cellular Fe^2+^, lower number of ballon-like cells, as well as increased GSH content and improved cell viability were observed in the LD + TOC group compared to the LD group (Fig. 4G–M). The protein expression of ACSL4, ALOX15, GPX4, SLC3A2, and SLC7A11 were also reversed by Tocilizumab (Fig. 4N–P). The role of IL-6 signaling in LPS/iE-DAP-induced STAT3 activation and ferroptosis was also verified by *IL6* knockdown. Consistent with the results of TOC experiments, the knockdown of *IL6* significantly reversed changes in lipid ROS, cell viability, cellular Fe^2+^, GSH content, and MDA formation in the si-*IL6* + LD group compared to the si-NC + LD group (Figs. S6A–F). Additionally, *IL6* knockdown decreased protein expression of ACSL4, ALOX15, and p-STAT3 and increased protein expression of GPX4, SLC3A2, and SLC7A11 (Figs. S6G–H). These findings proved that the IL-6/STAT3 signaling pathway mediated LPS/iE-DAP-induced ferroptosis.

### 3.5 Activated ferritinophagy by IL-6/STAT3 signaling contributed to LPS/iE-DAP-induced ferroptotic cell death

**Fig. 5:**
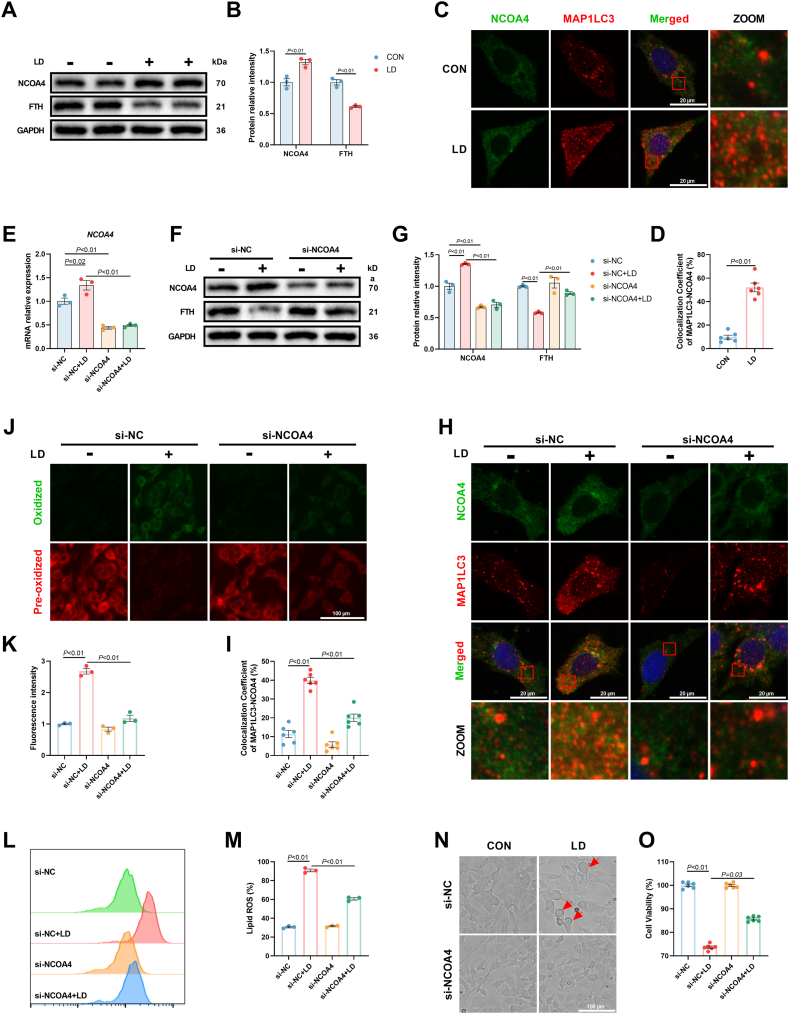
***NCOA4* knockdown blocked LPS/iE-DAP-induced ferritinophagy.** (A–B) The protein expression of FTH and NCOA4, and (C–D) colocalization analysis of MAP1LC3 and NCOA4 in hepatocytes with or without LPS/iE-DAP treatment; n = 3–6 per group. (E) Gene expression of *NCOA4*, (F–G) protein expression of NCOA4 and FTH in indicated groups, and (H–I) colocalization analysis of MAP1LC3 and NCOA4 in indicated groups; n = 3 per group. (J–M) Fluorescence images of lipid ROS observed by confocal microscope (Scale bar = 100 μm) and proportion of lipid ROS positive cells analyzed by flow cytometer; n = 3 per group. (N) Cell morphology was observed through phase-contrast pattern of optical microscope and red arrow indicates ballooning cells, scale bar = 100 μm. (O) Cell viability was analyzed using a CCK-8 kit; n = 6 per group. Data are presented as mean ± SEM.

**Fig. 6:**
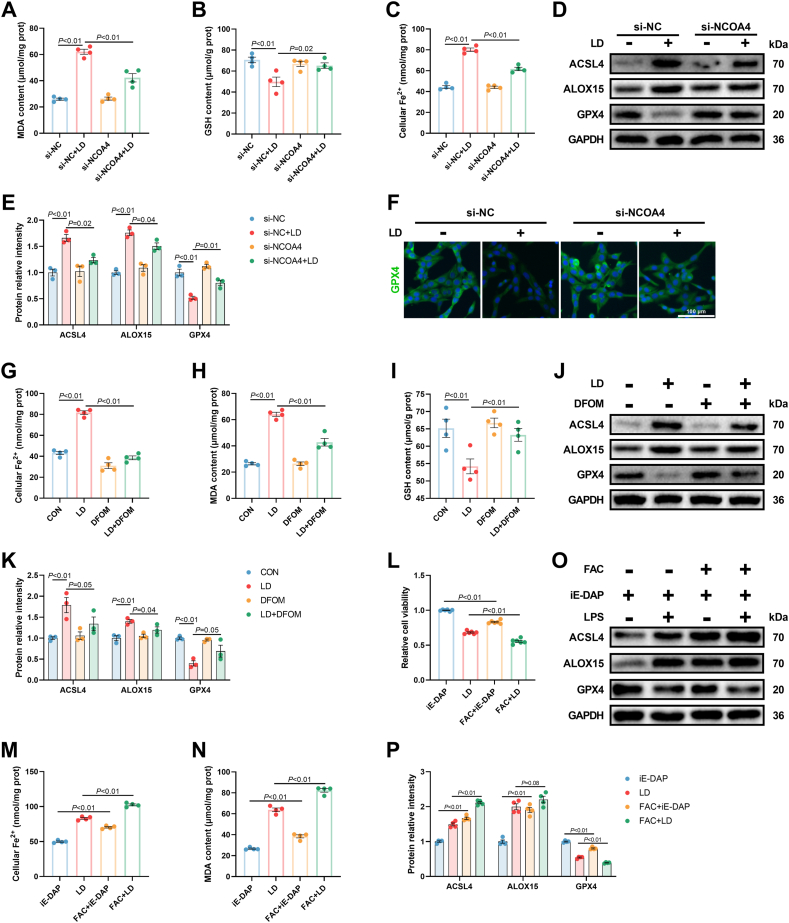
**Inhibition of ferritinophagy alleviated LPS/iE-DAP-induced ferroptotic cell death.** (A) MDA content, (B) GSH content, and (C) cellular Fe^2+^ in indicated groups; n = 4 per group. (D–E) Protein expression of ACSL4, ALOX15, and GPX4 in different groups; n = 3 per group. (F) Immunofluorescence analysis of GPX4, scale bar = 100 μm. (G) Cellular Fe^2+^, (H) MDA content, (I) GSH content, and (J–K) protein expression of ACSL4, ALOX15, and GPX4 in indicated groups; n = 3–4 per group. (L) Cell viability, (M) cellular Fe^2+^, (N) MDA content, and (O–P) protein expression of ACSL4, ALOX15, and GPX4 in indicated groups; n = 4–6 per group. Data are presented as mean ± SEM.

In addition to the JAK-STAT signaling pathway, KEGG enrichment analysis revealed a significant upregulation of the autophagy signaling pathway in LPS/iE-DAP-treated hepatocytes (Fig. 3C). Intriguingly, previous studies have shown that ferritinophagy is regulated by STAT3 and affected ferroptosis through controlling intracellular labile Fe^2+^ level [31]. Our study demonstrated that LPS/iE-DAP treatment decreased the protein expression of FTH (a component of ferritin) and increased the protein expression of NCOA4 (an autophagy-related protein that recognizes ferritin) (Fig. 5A and B). Immunofluorescence analysis revealed that LPS/iE-DAP led to more amounts of MAP1LC3 (a universal autophagy marker) particles, increased colocalization of MAP1LC3 with NCOA4 (Fig. 5C and D), as well as increased colocalization of FTH with LAMP1 (a lysosomal membrane protein) (Figs. S6I–J), representing enhanced ferritinophagy flux. Moreover, suppressing IL-6/STAT3 signaling through either *IL6* and *STAT3* knockdown or pharmacological intervention reversed LPS/iE-DAP-induced FTH degradation (Figs. S7A–D). TOC or Stattic treatment-induced ferritinophagy flux blockade was confirmed by decreased colocalization of MAP1LC3 with NCOA4 and LAMP1 with FTH (Figs. S7E–H), indicating that LPS/iE-DAP-induced ferritinophagy was regulated by IL-6/STAT3 signaling. To investigate whether ferritinophagy serves as a key intermediary between IL-6/STAT3 signaling and ferroptosis in LPS/iE-DAP-treated hepatocytes, *NCOA4* was knocked down to inhibit ferritinophagy. The efficiency of si-*NCOA4* was confirmed by decreased gene and protein expression of NCOA4 (Fig. 5E–G). *NCOA4* knockdown markedly increased the protein expression of FTH (Fig. 5F and G) and led to ferritinophagy flux blockade, as evidenced by decreased colocalization of MAP1LC3 with NCOA4 (Fig. 5H and I) and LAMP1 with FTH (Figs. S6K–L). Meanwhile, *NCOA4* knockdown decreased the number of balloon-like cells, lipid ROS content, MDA formation and cellular Fe^2+^ level, and increased cell viability and GSH content (Fig. 5, Fig. 6A–C). Besides, *NCOA4* knockdown also decreased protein expression of ACSL4 and ALOX15 while increasing protein expression of GPX4 (Fig. 6D and E). Consistent with the results of western blot analysis, immunofluorescence assay showed that *NCOA4* knockdown restored intracellular GPX4 level (Fig. 6F). Given that ferritinophagy contributes ferroptosis primarily by increasing intracellular Fe^2+^ level, thus we used deferoxamine mesylate (DFOM), an iron chelator, to decrease excessive cellular Fe^2+^. Similar to the results of *NCOA4* knockdown experiments, DFOM addition alleviated LPS/iE-DAP-induced ferroptosis with signs of decreased cellular Fe^2+^ and MDA formation, increased GSH content, decreased protein expression of ACSL4 and ALOX15, and increased expression of GPX4 (Fig. 6G–K). Moreover, using ferric ammonium citrate (FAC) to increase intracellular Fe^2+^ induced ferroptosis in hepatocytes treated with iE-DAP, as evidenced by decreased cell viability, increased MDA formation, decreased protein expression of GPX4, and increased protein expression of ACSL4 and ALOX15. Compared to iE-DAP-treated hepatocytes, the above indicators showed a similar variation trend in LPS/iE-DAP-treated hepatocytes challenged with or without FAC (Fig. 6L–P). These findings suggested that activated ferritinophagy by IL-6/STAT3 signaling contributed to LPS/iE-DAP-induced ferroptotic cell death.

### 3.6 Iron efflux blockade induced by elevated IL-6/STAT3 signaling also aggravated LPS/iE-DAP-induced ferroptotic cell death

**Fig. 7:**
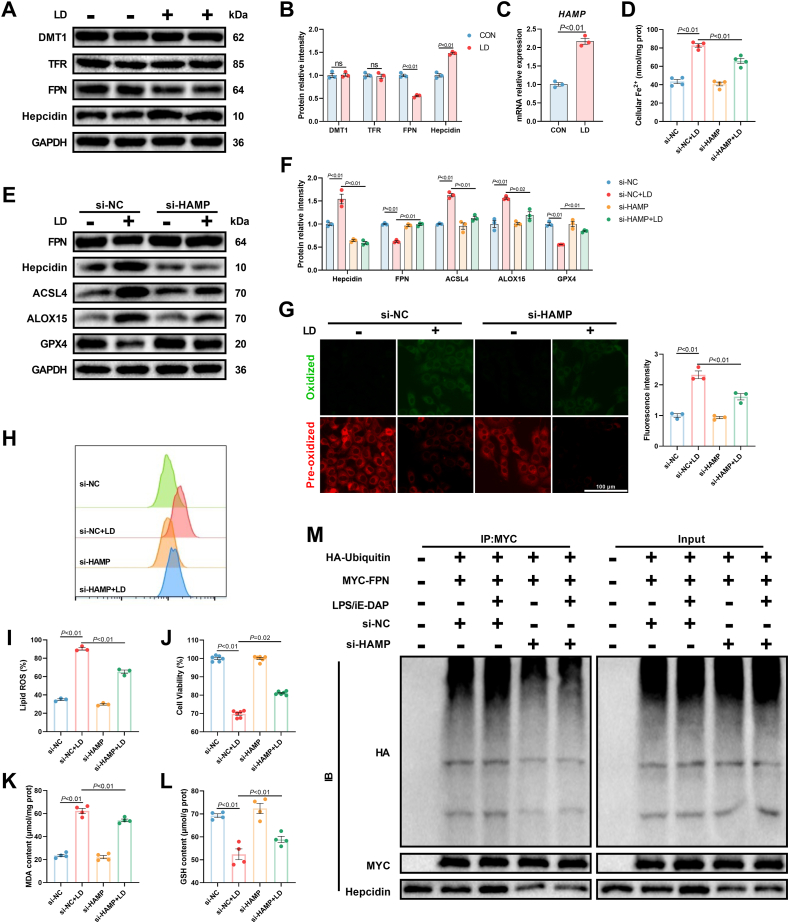
**Iron efflux blockade contributed to LPS/iE-DAP-induced ferroptotic cell death.** (A) Gene expression of *HAMP* and (B–C) protein expression of DMT1, TFR, FPN, and hepcidin in hepatocytes treated with or without LPS/iE-DAP; n = 6 per group. (D) Cellular Fe^2+^ content. (E–F) Protein expression of FPN, hepcidin, ACSL4, ALOX15, and GPX4 in indicated groups; n = 3 per group. (G–I) Fluorescence images of lipid ROS observed by confocal microscope (Scale bar = 100 μm) and proportion of lipid ROS positive cells analyzed by flow cytometer; n = 3 per group. (J) Cell viability, (K) MDA content, and (L) GSH content in different groups; n = 4–6 per group. (M) Coimmunoprecipitation and ubiquitination analysis of FPN in hepatocytes; n = 3 per group. Data are presented as mean ± SEM.

The homeostasis of intracellular iron depends not only on the intracellular signaling network but also on the close connection between the cell and the extracellular environment. This prompted us to explore whether cellular iron absorption and iron efflux were altered. *In vivo* study showed that in the livers of cows with ruminal dysbiosis, the hepatic iron absorption was not affected indicated by unchanged protein levels of DMT1 and TFR (Figs. S1L–M). However, hepatic iron efflux was impaired, as evidenced by increased protein expression of hepcidin and decreased protein expression of FPN (Figs. S1L–M). Similarly, in LPS/iE-DAP-treated hepatocytes, we observed increased gene expression of *HAMP* (the gene symbol of hepcidin), elevated protein expression of hepcidin, and decreased protein expression of FPN, while the protein expression of DMT1 and TFR was not significantly changed (Fig. 7A–C). This provoked a hypothesis that whether iron efflux blockade also contributed to Fe^2+^ accumulation and subsequently facilitated ferroptosis. To test this hypothesis, *HAMP* was knocked down and we found that protein expression of hepcidin and cellular Fe^2+^ were decreased while the protein expression of FPN was increased (Fig. 7D–F). As FPN is the sole known cellular exporter of elemental iron, knockdown of *HAMP* rescued iron efflux. Moreover, *HAMP* knockdown inhibited LPS/iE-DAP-induced ferroptotic cell death, as evidenced by increased cell viability and GSH content, decreased lipid ROS and MDA formation, inhibited protein expression of ACSL4 and ALOX15, and elevated protein expression of GPX4 (Fig. 7E–L). We next investigated how hepcidin decreased FPN expression. Previous studies have shown that elevated ubiquitination leads to proteasomal degradation of proteins [32]. To explore whether hepcidin influences the ubiquitination level of FPN, ubiquitin and FPN were overexpressed in hepatocytes, and co-immunoprecipitation assay was conducted. Western blot analysis revealed that LPS/iE-DAP increased the ubiquitination level of FPN; moreover, LPS/iE-DAP increased the coprecipitation level of hepcidin with anti-MYC antibody, suggesting a higher binding affinity between hepcidin and FPN (Fig. 7M). These results demonstrated that hepcidin mediated ubiquitination and degradation of FPN through direct interaction with FPN. This conclusion was further confirmed by the MG-132 experiment, in which MG-132 (a proteasome inhibitor) reversed LPS/iE-DAP-induced FPN degradation (Figs. S8A–B). Notably, MG-132 treatment significantly decreased cellular Fe^2+^ level (Fig. S8C), indicative of an effective cellular iron efflux function in the face of excessive cellular Fe^2+^ induced by enhanced ferritinophagy. To our expectation, *HAMP* knockdown decreased the binding affinity of hepcidin to FPN and abolished the ubiquitination degradation of FPN in LPS/iE-DAP-treated hepatocytes (Fig. 7M). Collectively, these data demonstrated that hepcidin-mediated ubiquitination degradation of FPN contributed to iron efflux blockade and intracellular Fe^2+^ overload, thereby aggravating LPS/iE-DAP-induced ferroptosis.

Notably, a previous study has reported that STAT3 could transcriptionally activate *HAMP* to degrade FPN, regulating iron metabolism [33]. We found that *STAT3* knockdown decreased gene expression of *HAMP*, indicating inhibited transcription level of *HAMP* (Fig. S8D). Besides, *STAT3* knockdown decreased protein expression of hepcidin, increased protein expression of FPN, inhibited the binding affinity of hepcidin to FPN, and suppressed the ubiquitination of FPN (Figs. S8E–G). Therefore, we identified that hepcidin-FPN-dependent iron efflux was regulated by IL-6/STAT3 signaling pathway in LPS/iE-DAP-treated hepatocytes.

### 3.7 Quercetin attenuated ferroptotic cell death in LPS/iE-DAP-treated hepatocytes by targeting IL-6/STAT3 signaling

**Fig. 8:**
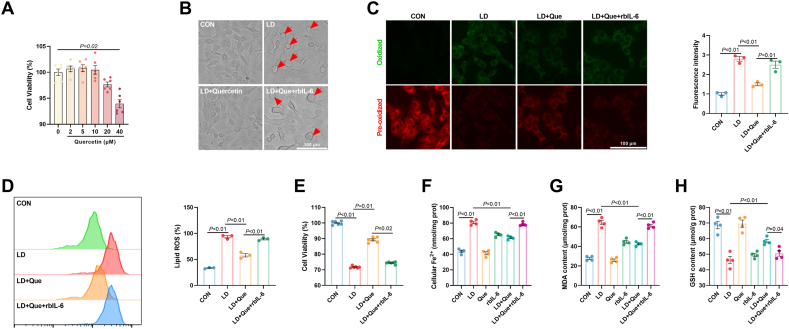
**Quercetin attenuated LPS/iE-DAP-induced ferroptotic cell death.** (A) Effect of different concentrations of quercetin on cell viability; n = 6 per group. (B) Cell morphology was observed through phase-contrast pattern of optical microscope and red arrow indicates ballooning cells, scale bar = 100 μm. (C–D) Fluorescence images of lipid ROS observed by confocal microscope (Scale bar = 100 μm) and proportion of lipid ROS positive cells analyzed by flow cytometer; n = 3 per group. (E) Cell viability, (F) cellular Fe^2+^, (G) MDA content, and (H) GSH content in indicated groups; n = 4–6 per group. Data are presented as mean ± SEM.

**Fig. 9:**
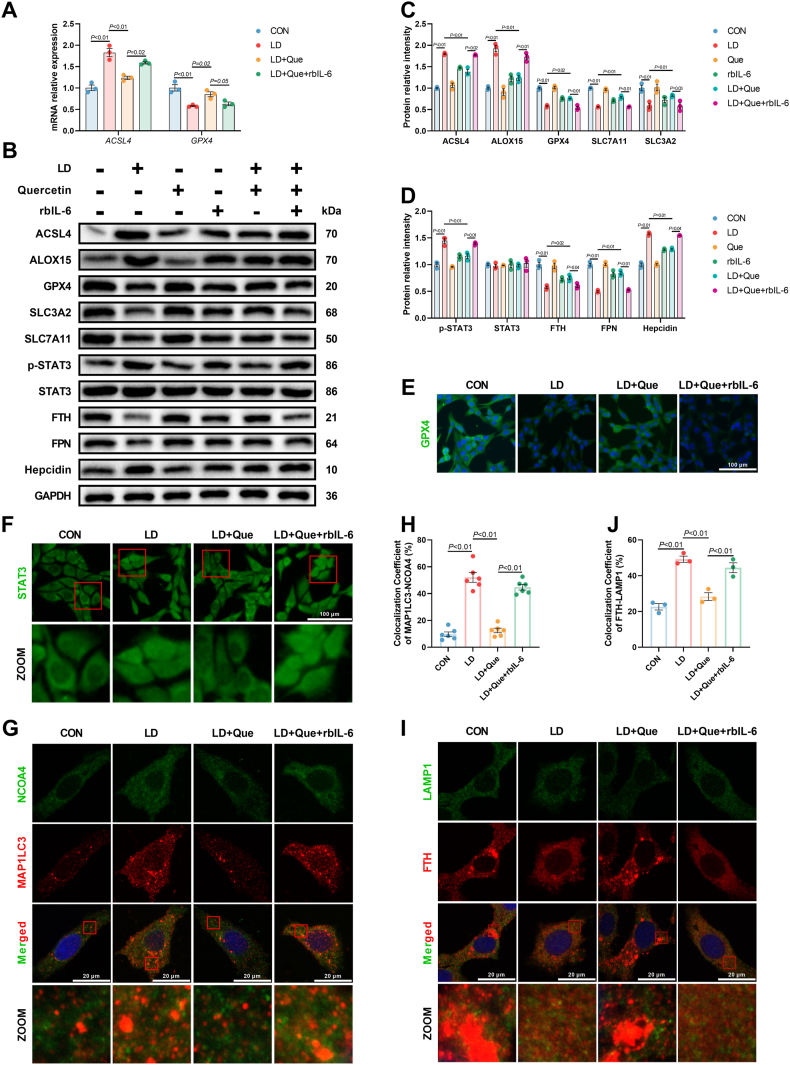
**Quercetin suppressed IL-6/STAT3 signaling pathway, blocked ferritinophagy flux, inhibited lipid peroxidation, and recovered system x_c_**^**-**^**-GPX4 in LPS/iE-DAP-treated hepatocytes.** (A) Gene expression of *ACSL4* and *GPX4*, and (B–D) protein expression of ACSL4, ALOX15, GPX4, SLC3A2, SLC7A11, p-STAT3, STAT3, FTH, FPN, and hepcidin in indicated groups; n = 3 per group. (E) Immunofluorescence analysis of GPX4, scale bar = 100 μm. (F) Cellular location of STAT3 was detected with immunofluorescence analysis, scale bar = 100 μm. (G–H) Colocalization analysis of MAP1LC3 and NCOA4, and (I–J) colocalization analysis of FTH and LAMP1 in indicated groups; n = 3–6 per group. Data are presented as mean ± SEM.

Given the inhibitory effect of quercetin on ferroptosis and its well-documented role in reducing the secretion of inflammatory cytokines, we selected quercetin to investigate whether it could alleviate LPS/iE-DAP-induced ferroptosis and hepatocyte injury. CCK8 assay was conducted to evaluate the cytotoxicity of different concentrations of quercetin (Que) on hepatocytes, and a noncytotoxic dose of quercetin (20 μM) was selected for subsequent experiments (Fig. 8A). Our study found that quercetin alleviated LPS/iE-DAP-induced ferroptotic cell death, which was verified by declines in the number of balloon-like cells, the content of lipid ROS, cellular Fe^2+^ and MDA formation, and increases in the cell viability and GSH content (Fig. 8B–H). Furthermore, quercetin pretreatment significantly decreased the gene expression of *ACSL4* and protein expression of ACSL4 and ALOX15, recovered gene expression of *GPX4* and protein expression of GPX, SLC7A11, and SLC3A2, and increased the fluorescence intensity of GPX4 (Fig. 9A–E). Besides, quercetin suppressed ferritinophagy and recovered iron efflux, as indicated by increased protein expression of FTH and FPN, decreased protein expression of hepcidin, as well as decreased colocalization of MAP1LC3 with NCOA4 and LAMP1 with FTH (Fig. 9B–D and Fig. 9G–J). Moreover, quercetin inactivated IL-6/STAT3 signaling with signs of decreased protein expression of p-STAT3 and translocation of STAT3 from the nucleus to the cytoplasm (Fig. 9B–D, and Fig. 9F). To investigate whether IL-6/STAT3 signaling was involved in the protective effect of quercetin against LPS/iE-DAP-induced hepatocyte injury and ferroptosis, recombinant bovine IL-6 (rbIL-6) was employed. Addition of rbIL-6 activated STAT3, increased hepcidin protein expression, decreased protein expression of FTH and FPN, and increased the colocalization of MAP1LC3 with NCOA4 as well as FTH with LAMP1 (Fig. 9), indicating suppressed iron efflux, and reactivated ferritinophagy flux. Meanwhile, rbIL-6 addition abolished the protective effects of quercetin, as shown by the increased number of balloon-like cells, elevated lipid ROS, cellular Fe^2+^ and MDA formation, decreased cell viability and GSH content, increased protein expression of ACSL4 and ALOX15, and decreased protein expression of GPX4, SLC3A2, and SLC7A11. (Fig. 8, Fig. 9B-C). Therefore, our results demonstrated that quercetin protected hepatocytes from LPS/iE-DAP-induced cell injury and ferroptosis by regulated IL-6/STAT3-dependent ferritinophagy and iron efflux.

### 3.8 Quercetin alleviated liver injury and ferroptosis in LPS/iE-DAP-challenged mice

**Fig. 10:**
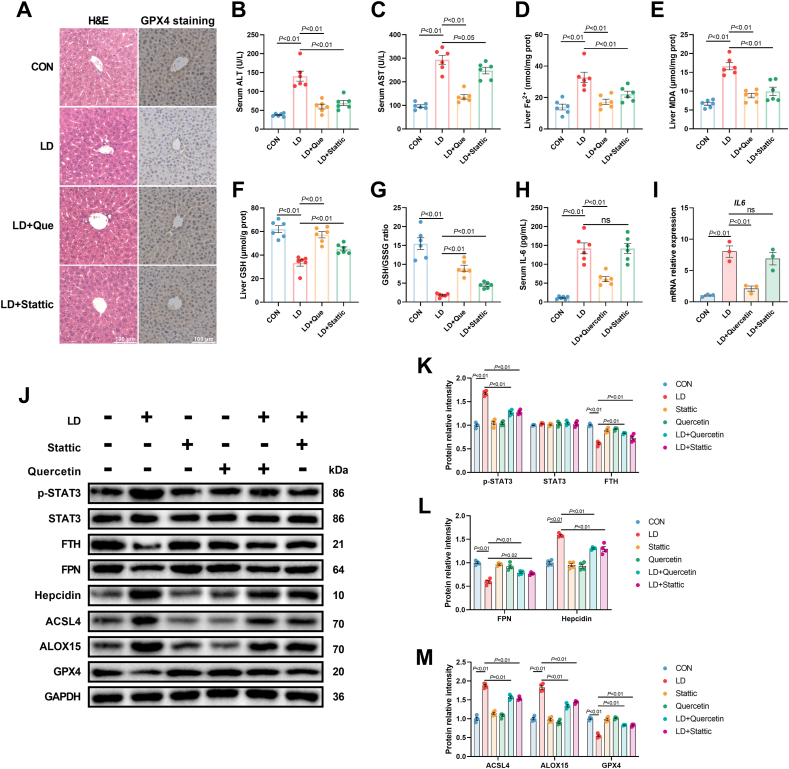
**Quercetin pretreatment alleviated liver injury and hepatic ferroptosis in LPS/iE-DAP-injected C57 mice.** (A) Hematoxylin eosin staining and immunohistochemistry analysis of liver section, scale bar = 100 μm. (B) Serum ALT level, (C) AST level, (D) hepatic Fe^2+^, (E) MDA content, (F) GSH content, (G) GSH/GSSG ratio, and (H) serum IL-6 level in indicated groups; n = 6 per group. (I) Gene expression of *IL6* and (J–M) protein expression of p-STAT3, STAT3, FTH, FPN, hepcidin, ACSL4, ALOX15, and GPX4 in the livers of different groups; n = 4 per group. Data are presented as mean ± SEM.

The protective effect of quercetin against LPS/iE-DAP-induced liver injury was further confirmed in C57 mice. As shown in Fig. 10, quercetin alleviated inflammatory cell infiltration and reversed the increased levels of ALT and AST induced by LPS/iE-DAP co-injection (Fig. 10A–C). Meanwhile, LPS/iE-DAP increased hepatic Fe^2+^, MDA content and decreased hepatic GSH content and GSH/GSSG ratio, all of which were reversed by quercetin (Fig. 10D–G). Immunohistochemistry results showed that LPS/iE-DAP reversed the decreased GPX4 content induced by LPS/iE-DAP injection (Fig. 10A). Further analysis revealed that quercetin decreased the gene expression of *IL6* and serum IL-6 content, increased protein expression of FTH and FPN, and decreased protein expression of p-STAT3 and hepcidin compared to those in the LD group (Fig. 10H–L). These results suggest that quercetin inactivated the IL-6/STAT3 signaling, suppressed ferritinophagy, and restored iron efflux. Besides, quercetin decreased protein expression of ACSL4 and ALOX15, and increased protein expression of GPX4 (Fig. 10J and M). Of note, injection of Stattic showed a similar effect to quercetin on most of the above indices except gene expression of *IL6* and serum IL-6 content (Fig. 10). Based on these findings, we concluded that quercetin protected against LPS/iE-DAP-induced liver injury and ferroptosis by regulating the IL-6/STAT3 pathway *in vivo*.

## 4 Discussion

Ruminal dysbiosis is prevalent in dairy cows and its incidence has been reported to reach 30 % in some dairy herds [34]. The liver injury caused by ruminal dysbiosis brings a great burden to dairy farms, as it not only reduces milk yield but also results in secondary damage to other organs [1,35]. Therefore, investigating the underlying mechanism of ruminal dysbiosis-derived liver injury and finding the corresponding solution to mitigate this liver injury are necessary. LPS and iE-DAP are two pathogenic metabolites in the rumen. Previous studies mostly focused on the effects of LPS on ruminal dysbiosis-induced liver injury, while the role of iE-DAP has been largely ignored. Therefore, the present study used LPS and iE-DAP to establish *in vitro* and *in vivo* liver injury models. Our findings show that iE-DAP exacerbates LPS-induced liver and hepatocyte injury and verify that ferroptosis mediates LPS/iE-DAP-induced liver and hepatocyte injury. To our knowledge, this is the first study demonstrating the effect of iE-DAP on ferroptotic cell death. Mechanistically, on one hand, STAT3, activated by IL-6, enhances ferritinophagy, resulting in increased intracellular free Fe^2+^; on the other hand, activated STAT3 suppresses iron efflux via the hepcidin/FPN pathway, thereby leading to intracellular iron accumulation. The consequent accumulation of intracellular Fe^2+^ triggers lipid peroxidation and impairs system x_c_^−^-GPX4 signaling, ultimately leading to ferroptotic cell death. Furthermore, our study demonstrates that quercetin could alleviate LPS/iE-DAP-induced hepatocyte and liver injury, as well as ferroptosis, by regulating the IL-6/STAT3 signaling pathway, which provides a theoretical basis for treating liver injury in dairy cows.

Ferroptosis is an iron-dependent programmed cell death and is considered an important driver of many diseases. The liver functions as a metabolism center and the major regulator of systemic iron homeostasis [36], which forms an innate condition for hepatic ferroptosis when the liver is exposed to pathogenic metabolites. Ferroptosis is driven by two factors: unrestrained lipid peroxidation and disruption of the system x_c_^−^ cystine-GSH-GPX4 pathway. Phospholipid hydroperoxides (PLOOHs), a type of lipid ROS, interact with Fe^2+^ to produce free radicals, including phospholipid peroxyl radicals (PLOO^▪^) and alkoxyl phospholipid radicals (PLO^▪^) [37]. These radicals react with polyunsaturated fatty acid phospholipids (PUFA-PLs) present in organelle membranes and cell membranes, initiating a damaging lipid peroxidation chain reaction and ultimately leading to membrane disruption and subsequent cell death [38]. The development of lipid peroxidation depends on various regulators. ACSL4 enables long-chain PUFA to bind with coenzyme A. These products can be esterified into phospholipids and absorbed as components of cell membranes [38]. Therefore, this biological process catalyzed by ACSL4 provides substrates for lipid peroxidation. Certain lipoxygenases, such as ALOX15, can directly oxygenate PUFA in cell membranes, triggering lipid peroxidation [39]. As the most important defender against ferroptosis, GPX4 catalyzes the reduction and detoxification of lipid peroxides [40]. GSH is considered a cofactor of GPX4 and its synthesis depends on system x_c_^−^ cystine/glutamate antiporter composed of SLC7A11 and SLC3A2 [41]. Loss of GPX4 leads to ferroptosis, which is reversed by ACSL4 deficiency [42]. In our study, we found that LPS treatment or LPS/iE-DAP co-treatment both increase cellular Fe^2+^. We also observed an increased number of balloon-like hepatocytes and damaged mitochondria, which were the typical morphology of ferroptotic cells. Our findings showed that the expression of ACSL4 and ALOX15 was upregulated while the expression of GPX4, SLC7A11, and SLC3A2 was downregulated with LPS treatment or LPS/iE-DAP co-treatment, indicating impaired system x_c_^−^ cystine-GSH-GPX4 pathway and increased lipid peroxidation. Intriguingly, the iE-DAP used in this experiment was noncytotoxic and did not significantly influence lipid peroxidation or system x_c_^−^ cystine-GSH-GPX4 pathway, but could promote LPS-induced lipid peroxidation, GPX4 impairment, and ferroptotic cell death. Two hypotheses were proposed for this phenomenon: (I) Although iE-DAP increased intracellular Fe^2+^ content, this increase was not sufficient to initiate lipid peroxidation; (II) iE-DAP-caused slight increase in intracellular Fe^2+^ content led to limited lipid peroxidation, which can be immediately interrupted by the powerful system x_c_^−^ cystine-GSH-GPX4. To verify these hypotheses, *GPX*4 was knocked down and we found that in both iE-DAP-treated hepatocytes and LPS/iE-DAP-treated hepatocytes, lipid peroxidation and ferroptosis levels were upregulated (Figs. S2G–N). These findings indicate that iE-DAP indeed triggers lipid peroxidation, which is reversed by system x_c_^−^ cystine-GSH-GPX4. However, in the condition of LPS challenge, system x_c_^−^ cystine-GSH-GPX4 is impaired, and the increased Fe^2+^ caused by iE-DAP could further promote the lipid peroxidation reaction, therefore aggravating ferroptotic cell death. Ferroptosis has been reported to be implicated in the pathogenesis of various liver diseases, including chronic liver injury, liver fibrosis, steatohepatitis, and hepatocellular carcinoma [36,43]. It was reported that ferroptosis was involved in acetaminophen-induced hepatic injury and using ferroptosis inhibitor Fer-1 had a moderate therapeutic effect [44]. This raises the question whether ferroptosis contributes to LPS/iE-DAP-induced hepatocyte injury? To this end, we used Fer-1 in hepatocytes to suppress lipid peroxidation and found that ferroptosis as well as decreased cell viability was alleviated. Fer-1 was also applied in mice and improved liver injury as well as alleviated hepatic ferroptosis was observed, providing strong evidence that ferroptosis mediates hepatocyte and liver injury induced by LPS/iE-DAP.

Over the years, a growing number of LPS-targeted signaling pathways have been reported. Although the studies on iE-DAP are not as much as studies on LPS, both LPS and iE-DAP can activate NF-κB and lead to transcription of its targeted cytokines genes [7]. In the present study, we identified IL-6/STAT3 signaling as a crucial regulator of LPS/iE-DAP-induced ferroptotic cell death, which was confirmed through inhibition of IL-6 and STAT3. Mechanistically, IL-6/STAT3 regulates ferroptosis via the following ways: (I) IL-6/STAT3/ferritinophagy-dependent ferritin degradation, and (II) IL-6/STAT3/hepcidin/FPN-dependent intracellular iron efflux blockage. Intracellular iron metabolism is a complex process that can be broadly categorized into three processes: iron absorption, iron storage and utilization, and iron export. Disorders in any of these processes can lead to the breakdown of cellular iron homeostasis. Most extracellular Fe^3+^ is bound to transferrin, which is recognized by the transferrin receptor 1 on the cell surface [18]. Then, Fe^3+^ is endocytosed into the cell via DMT1, where it is converted into Fe^2+^, to participate in numerous biological processes. Excessive cellular Fe^2+^ can be stored in ferritin, which is degraded through NCOA4-mediated ferritinophagy [45,46]. Enhanced ferritinophagy leads to the release of Fe^2+^ and provides substrates for lipid peroxidation, facilitating the occurrence of ferroptosis. In liver damage caused by cadmium and arsenic, increased levels of ferritinophagy and ferroptosis were found while inhibiting ferritinopagy alleviated ferroptosis and liver damage [47,48]. Moreover, ferritinophagy and ferroptosis were also implicated in the liver fibrosis [49]. The role of ferritinophagy in promoting ferroptosis was confirmed in our study through knockdown of *NCOA4*, a cargo receptor located in the autophagosome that facilitates autophagic turnover of ferritin [50]. Since ferritinophagy primarily promotes ferroptosis by increasing intracellular free Fe^2+^, we used an iron chelator, DFOM, to decrease cellular Fe^2+^ without affecting ferritinophagy. DFOM intervention produced similar effects with ferritinophagy inhibition on alleviating ferroptosis. However, the precise mechanism by which STAT3 regulates ferritinophagy remains unclear. Transcriptional activation of ferritinophagy-related genes by STAT3 may be a potential mechanism, which needs to be confirmed in further study. FPN is located on the cell surface and is the only known cellular exporter of elemental iron, and its activity is regulated by hepcidin [51]. Hepcidin binds to FPN and mediates ubiquitination of FPN, resulting in FPN endocytosis and eventual degradation [51]. Systemic inflammatory responses inhibit the absorption of iron from food into the bloodstream by duodenal enterocytes and the release of iron from macrophages into the bloodstream, leading to hypoferremia, in which STAT3 plays a vital role [33]. STAT3 can transcriptionally regulate *HAMP* and therefore indirectly control the activity of FPN. While the role of hepcidin on cellular iron homeostasis has been well understood, whether STAT3 can regulate ferroptosis through hepcidin/FPN signaling has not been reported. Our study found that the knockdown of *HAMP* or *STAT3* restored FPN to normal level, alleviating intracellular iron accumulation and ferroptotic cell death. Specifically, activated STAT3 increases the transcriptional expression of *HAMP* and promotes the binding of hepcidin to FPN, causing the ubiquitination degradation of FPN and consequent iron efflux blockade. Ferritinophagy and iron export are two separate processes, but under LPS/iE-DAP treatment, these processes are linked by IL-6/STAT3 signaling to promote intracellular iron accumulation and the onset of ferroptosis. Intriguingly, iron absorption was not affected in both hepatocytes and cow livers. Previous study suggests that under the inflammatory environment, levels of TFR and DMT1 are elevated and the iron absorption capacity is enhanced in microglia [52]. This inconsistency may be due to the difference in species and tissue. Our study is the first to reveal that STAT3 mediates ferroptosis through hepcidin/FPN signaling-dependent iron efflux blockade. Another innovation of this study is the comprehensive *in vivo* and *in vitro* analysis of the effects of iron metabolism on ferroptotic cell death from the perspectives of iron absorption, iron storage, and iron efflux.

Following the identification of the mechanism underlying LPS/iE-DAP-induced hepatocyte injury, we next sought solutions to mitigate this liver injury. Our previous study applied sodium butyrate to treat ruminal dysbiosis-derived liver injury [53]. The active ingredient of sodium butyrate is butyric acid, a short-chain fatty acid that has the effects of regulating gut microbiota, anti-oxidant, and anti-inflammatory [54,55]. However, the mechanisms whereby sodium butyrate alleviates ruminal dysbiosis-derived liver injury appear to be primarily through the regulation of ruminal microbiota rather than its anti-oxidant effects, and there are few studies about sodium butyrate inhibiting ferroptosis. The candidate should meet the following requirements: having an inhibitory effect on hepatic ferroptosis and could be applied as a feed additive to farms. Numerous studies have demonstrated the inhibitory effects of quercetin on ferroptosis. It has been reported that quercetin can inhibit ferroptosis and alleviate acute kidney injury [56], liver injury [57], and intestinal damage [22]. The anti-inflammatory and anti-oxidant properties of quercetin have also been widely acknowledged [58,59]. Moreover, the wide sources and low price of quercetin makes it available to be a feed additive [60,61]. In our *in vivo* and *in vitro* studies, we found that hepatocyte and liver injury, ferroptosis, increased ferritinophagy, and blocked iron export were all reversed by quercetin intervention. Additionally, IL-6/STAT3 signaling pathway was inhibited by quercetin. To confirm whether the effect of quercetin on ferroptosis was mediated through IL-6/STAT3 signaling, we treated hepatocytes with rbIL-6 and observed that rbIL-6 addition activated STAT3 and abolished the alleviating effects of quercetin on ferroptosis and hepatocyte injury. Furthermore, intraperitoneal injection of STAT3 inhibitor Stattic in mice showed similar effects to quercetin on liver injury and ferroptosis. We believe that after being ingested by animals, quercetin is absorbed through the digestive tract and reaches the liver, where it plays a role in resisting ferroptosis, thereby alleviating ruminal dysbiosis-derived liver injury. However, due to the limitations in purchasing and feeding dairy cows, we did not investigate whether quercetin could also prevent ruminal dysbiosis-induced liver injury and hepatic ferroptosis in cows. Overall, these findings support the conclusion that quercetin protects against 10.13039/501100012274LPS/iE-DAP-induced liver injury and ferroptosis by regulating ferritinophagy and cellular iron export via the IL-6/STAT3 signaling pathway, a mechanism not previously reported.

In conclusion, a novel model of liver injury was established by the combination of LPS and iE-DAP in our study. Our findings revealed that addition of a noncytotoxic dose of iE-DAP aggravated LPS-induced hepatocyte injury, which was mediated by ferroptosis. Mechanistically, LPS/iE-DAP treatment increased IL-6 level and activated STAT3, thus leading to enhanced ferritinophagy and intracellular iron efflux blockade. This disruption in iron homeostasis resulted in cellular iron accumulation, increased lipid peroxidation, and ultimately triggered ferroptotic cell death. Moreover, we demonstrated that quercetin, a natural compound, could alleviate LPS/iE-DAP-induced liver injury and ferroptosis by regulating IL-6/STAT3-dependent ferritinophagy and hepcidin/FPN-dependent iron efflux *in vivo* and *in vitro*. Collectively, our study exhibits a novel perspective on the mechanism whereby liver injury occurs and provides a potential solution for liver injury in cows with ruminal dysbiosis.

## CRediT authorship contribution statement

**Hongzhu Zhang:** Writing – review & editing, Writing – original draft, Methodology, Investigation, Formal analysis, Conceptualization. **Huimin Shi:** Methodology. **Xuerui Li:** Methodology. **Shendong Zhou:** Methodology. **Xiaokun Song:** Methodology. **Nana Ma:** Writing – review & editing. **Meijuan Meng:** Writing – review & editing. **Guangjun Chang:** Funding acquisition. **Xiangzhen Shen:** Supervision, Funding acquisition.

## Data availability

The data in the current study is available from the corresponding author upon reasonable request.

## Declaration of competing interest

The authors declare no competing interest.

## Funding

This study was financially supported by the 10.13039/501100001809National Natural Science Foundation of China (Grant 32172933 and 32373087; Beijing, China), the Key R&D Program of Ningxia Hui Autonomous Region of China (Grant 21BEF02019, Yinchuan, China), and 10.13039/501100004772Natural Science Foundation of Ningxia Hui Autonomous Region (Grant 2022AAC02072; Ningxia, China).

## Declaration of competing interest

The authors declare no competing interest.

## Data Availability

Data will be made available on request.
