# Supplementary material for: Quercetin alleviates LPS/iE-DAP-induced liver injury by suppressing ferroptosis via regulating ferritinophagy and intracellular iron efflux
Source: Redox Biol. 2025 Feb 18;81:103557. doi: 10.1016/j.redox.2025.103557 (PMC11904602; doi:10.1016/j.redox.2025.103557)
Supplement: Multimedia component 1 [file mmc1.docx]

**Supplementary Table 1.** The primers used in RT-qPCR

| **Gene** | **Primers (5´-3´)** | **Production length (bp)** | **Gene bank number** |
| --- | --- | --- | --- |
| **Bovine** | | | |
| *ACSL4* | AATTCATGACAAGCCAAACCC | 105 | XM_015461586.3 |
|  | ACAGAATAATCCTCAGCCGTT |  |  |
| *GPX4* | TCCTGGCCTTCCCTTGCAAC | 123 | NM_001346430.1 |
|  | CCCCATTTACACAGATCTTGCT |  |  |
| *NCOA4* | CGTTACTCTTTGAAGCCGACA | 228 | NM_001075868.1 |
|  | CAACGGCAGGTTTACTTCCA |  |  |
| *HAMP* | TGCTGCAGGACATAGACCAC | 113 | NM_001114508.2 |
|  | ACCAGCCATTTTATTTCAAGACT |  |  |
| *STAT3* | AGATTGCTGGTTAAATTCCCT | 109 | NM_001012671.2 |
|  | TGTTAAATTTCCGGGACCCTC |  |  |
| *IL6* | GGAGGAAAAGGACGGATGCT | 227 | NM_173923.2 |
|  | GGTCAGTGTTTGTGGCTGGA |  |  |
| **Mouse** | | | |
| *IL6* | GACTTCCATCCAGTTGCCTT | 150 | NM_001314054.1 |
|  | ATGTGTAATTAAGCCTCCGACT |  |  |


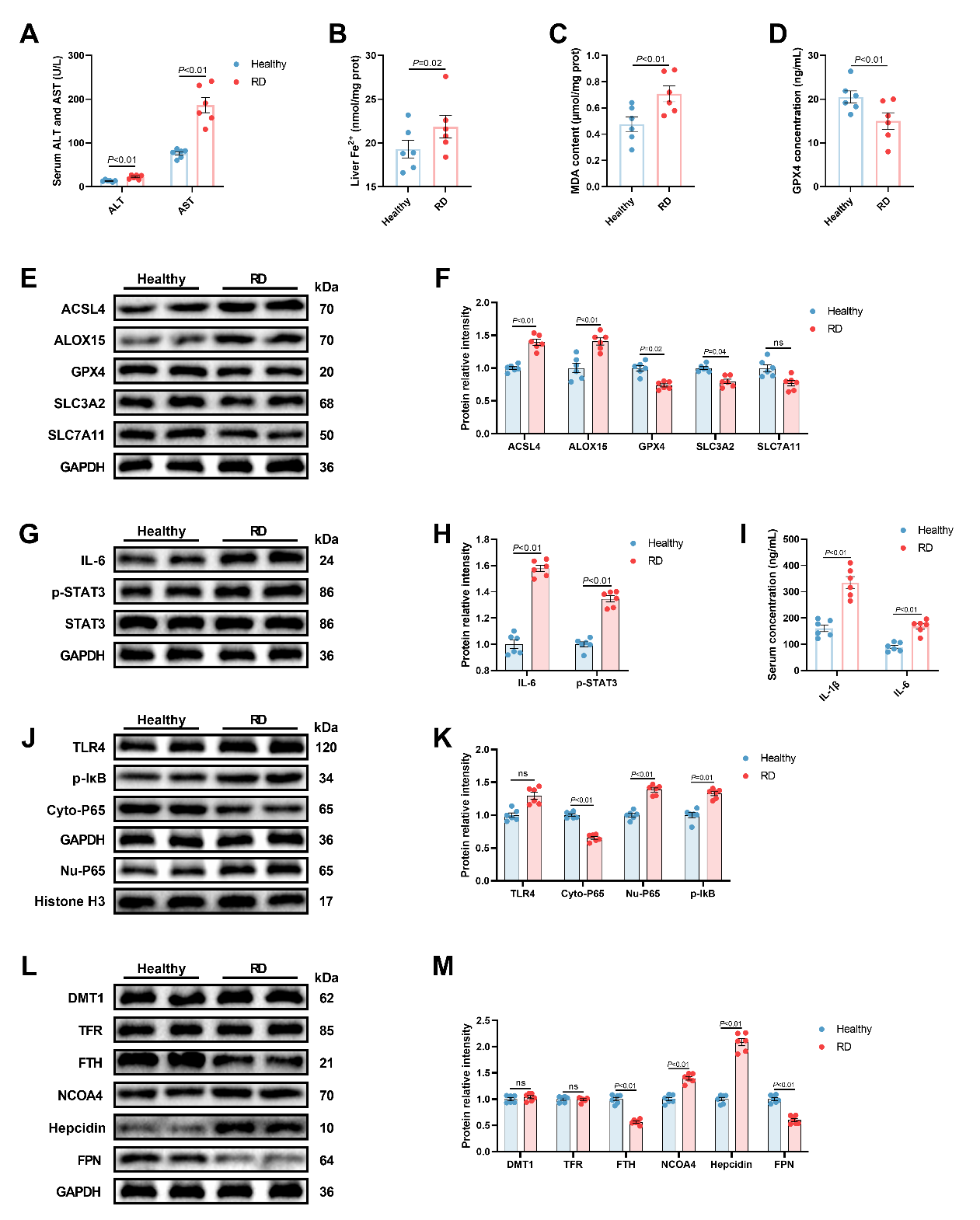


**Fig S1. Cows with ruminal dysbiosis showed liver injury and increased level of ferroptosis.** (A) Serum levels of ALT and AST, (B) hepatic Fe^2+^ content, (C) MDA content, and (D) serum level of GPX4 in healthy group and RD group; n = 6 per group. (E-H) Protein expression of ACSL4, ALOX15, GPX4, SLC3A2, SLC7A11, IL-6, p-STAT3, and STAT3 in indicated groups; n = 6 per group. (I) Serum concentration IL-1β and IL-6; n = 6 per group. (J-M) Protein expression of TLR4, cyto-P65, nu-P65, p-IκB, DMT1, TFR, FTH, NCOA4, hepcidin, and FPN in different groups; n = 6 per group. Data are presented as mean ± SEM. RD, ruminal dysbiosis.


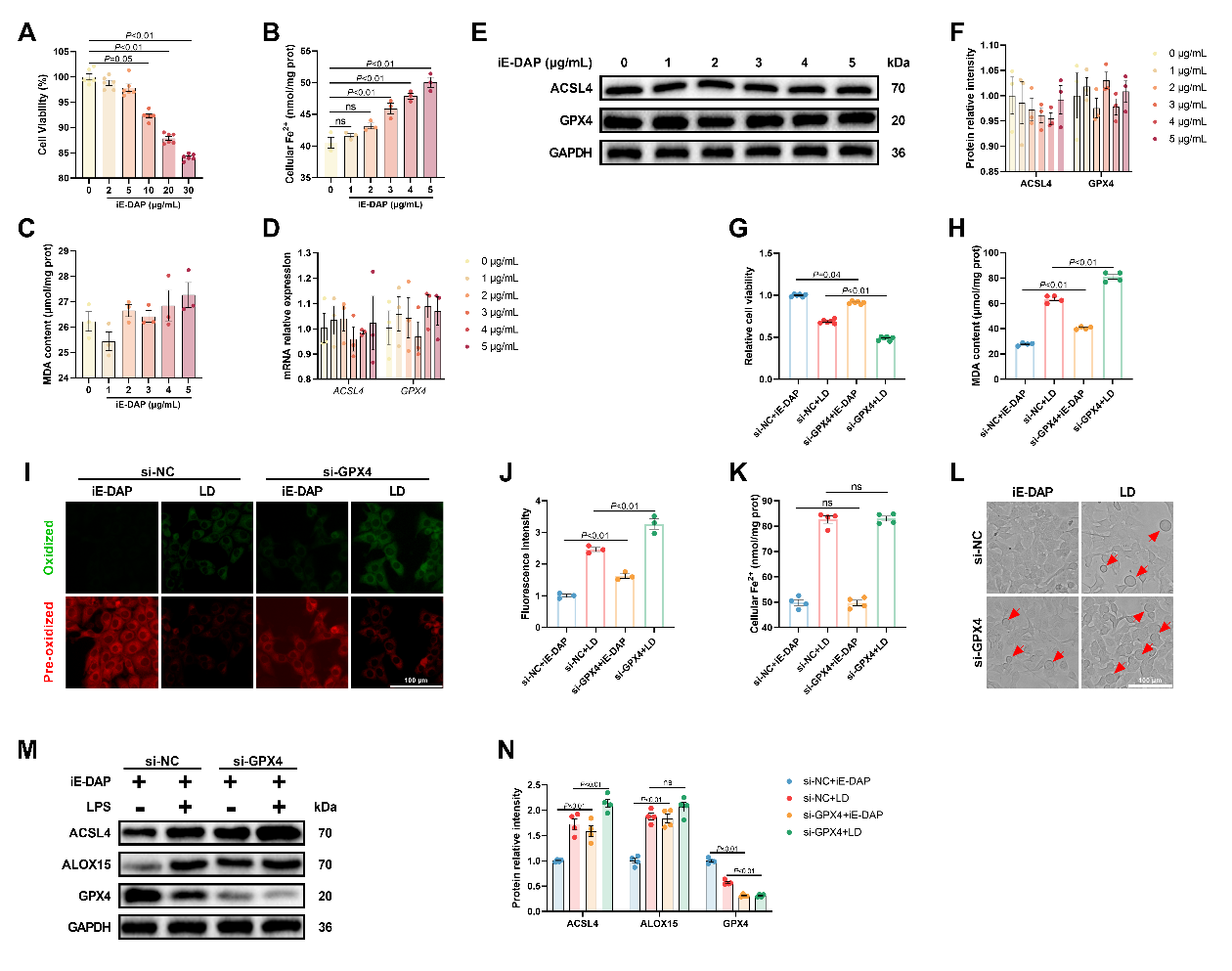


**Fig S2. Effect of different concentrations of iE-DAP on ferroptosis.** (A) Effect of different concentrations of iE-DAP on cell viability; n = 6 per group. (B) Cellular Fe^2+^ and (C) MDA content in indicated groups; n = 3 per group. (D-F) mRNA and protein expression of ACSL4 and GPX4 in hepatocytes treated with different dose of iE-DAP; n = 3 per group. (G) Cell viability, (H) MDA content, (I-J) Fluorescence images of lipid ROS, and (K) cellular Fe^2+^ in hepatocytes of different groups; n = 3-6 per group. (L) Cell morphology was observed through phase-contrast pattern of optical microscope and red arrow indicates ballooning cells, scale bar = 100 μm. (M-N) Protein expression of ACSL4, ALOX15, and GPX4 in hepatocytes of different groups; n = 4 per group. Data are presented as mean ± SEM.


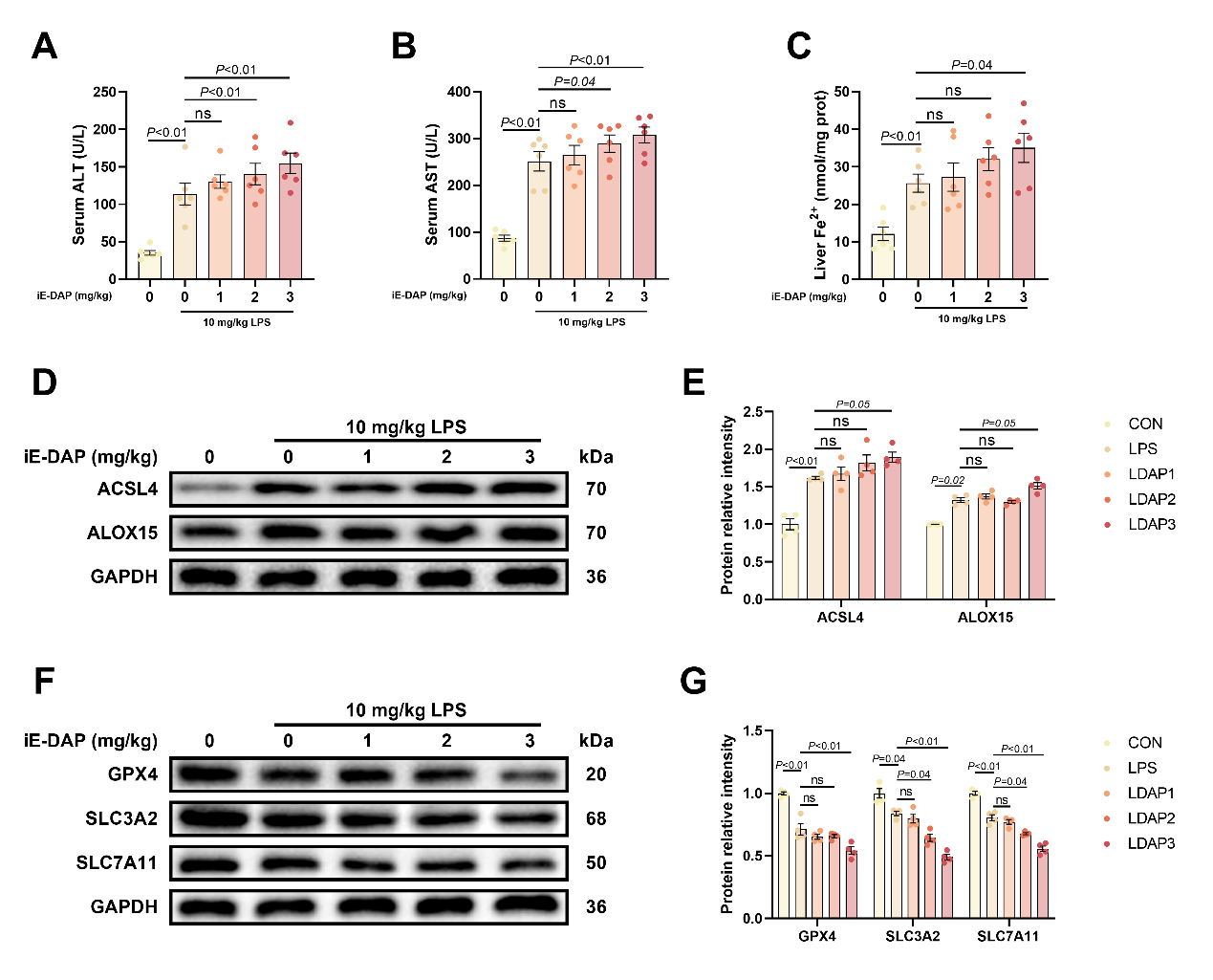


**Fig S3. Establishment of *in vivo* liver injury model induced by LPS and iE-DAP co-injection.** (A-B) Serum levels of ALT and AST, (C) hepatic Fe^2+^ content, and (D-G) protein expression of ACSL4, ALOX15, GPX4, SLC3A2, and SLC7A11 in different groups; n = 4-6 per group. Data are presented as mean ± SEM. LPS, 10 mg/kg LPS; LDAP1, 10 mg/kg LPS + 1 mg/kg iE-DAP; LDAP2, 10 mg/kg LPS + 2 mg/kg iE-DAP; LDAP3, 10 mg/kg LPS + 3 mg/kg iE-DAP.


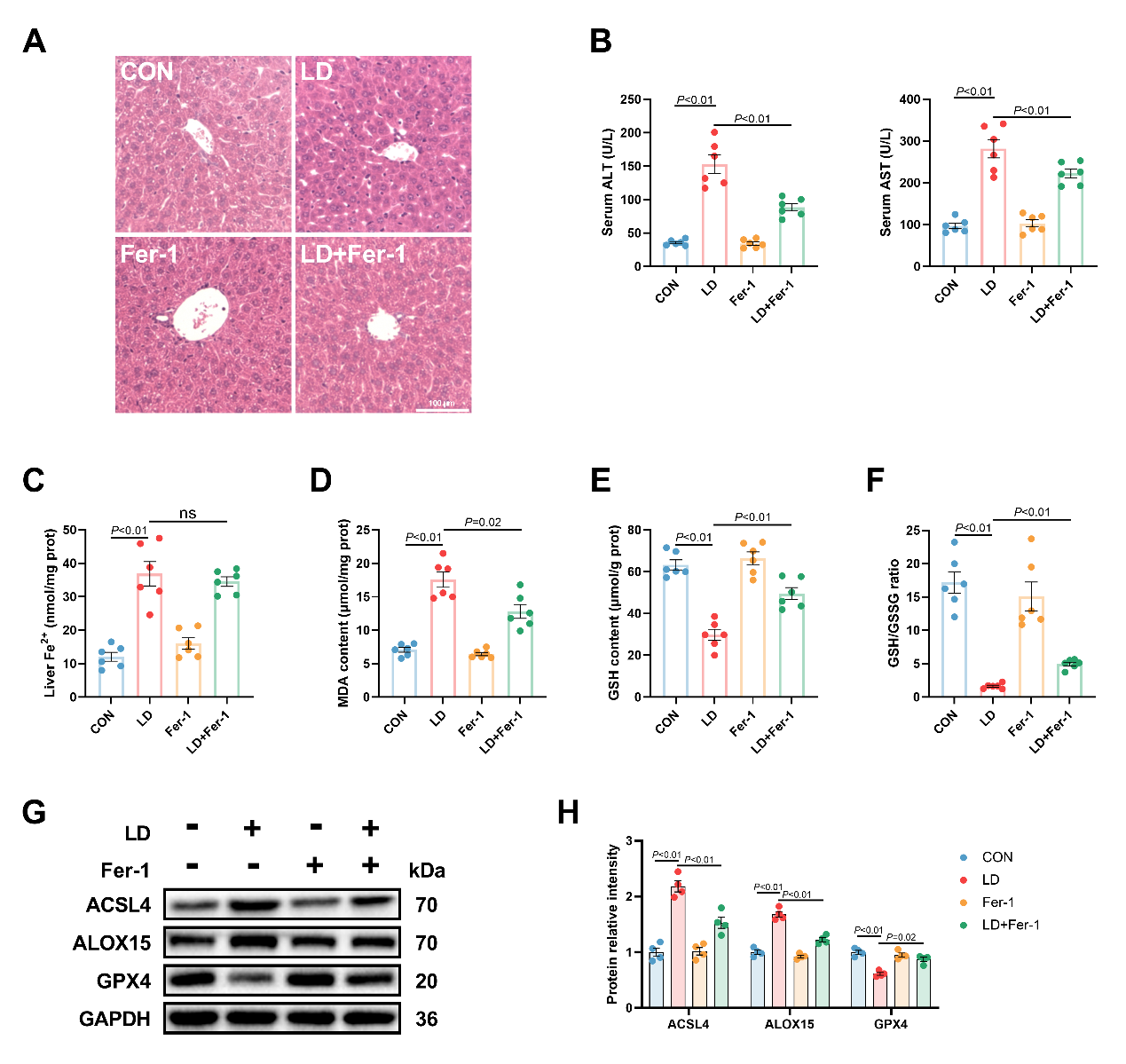


**Fig S4. Fer-1 intervention alleviated LPS/iE-DAP-induced liver injury and hepatic ferroptosis.** (A) Hematoxylin eosin staining. (B) Serum levels of ALT and AST, (C) Hepatic Fe^2+^ content, (D) MDA content, (E) GSH content, and (F) GSH/GSSG in indicated groups; n = 6 per group. (G-H) Protein expression of ACSL4, ALOX15, and GPX4 in the livers of different groups; n = 4 per group. Data are presented as mean ± SEM. LD, 10 mg/kg LPS + 3 mg/kg iE-DAP.


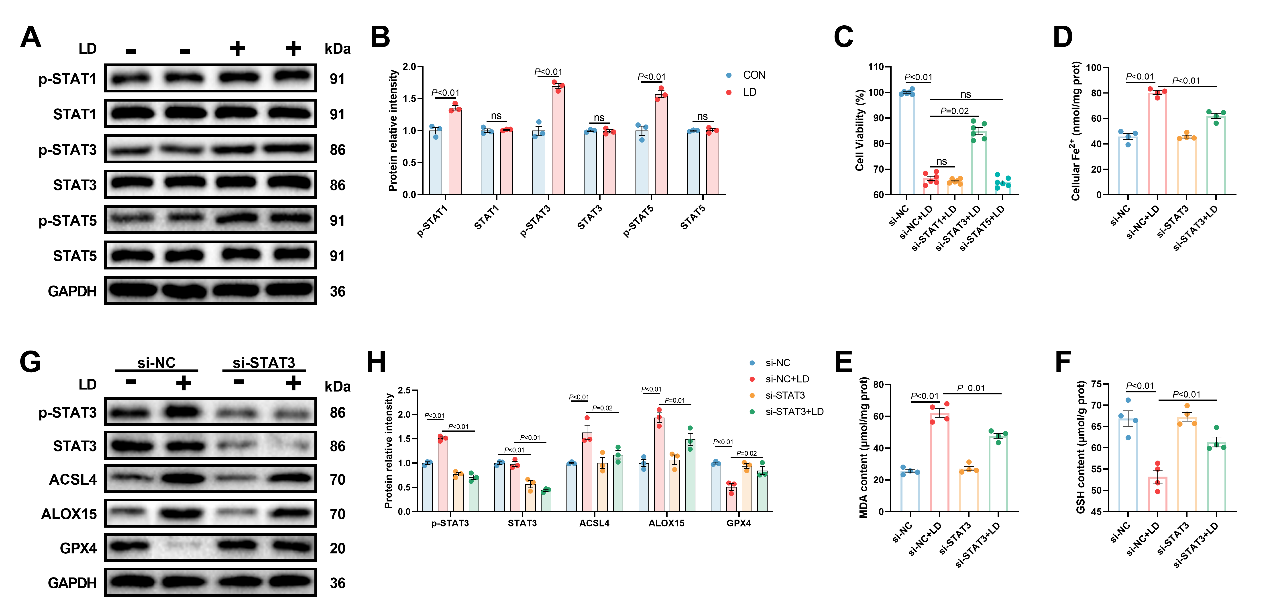


**Fig S5. Inhibition of IL-6/STAT3 signaling counteracted ferroptosis in LPS/iE-DAP-treated hepatocytes.** (A-B) Protein expression of p-STAT1, STAT1, p-STAT3, STAT3, p-STAT5, and STAT5 in hepatocytes treated with or without LPS/iE-DAP; n = 3 per group. (C) Cell viability, (D) cellular Fe^2+^, (E) MDA content, (F) GSH content, and (G-H) protein expression of p-STAT3, STAT3, ACSL4, ALOX15, and GPX4 in hepatocytes of different groups; n = 3-6. Data are presented as mean ± SEM.


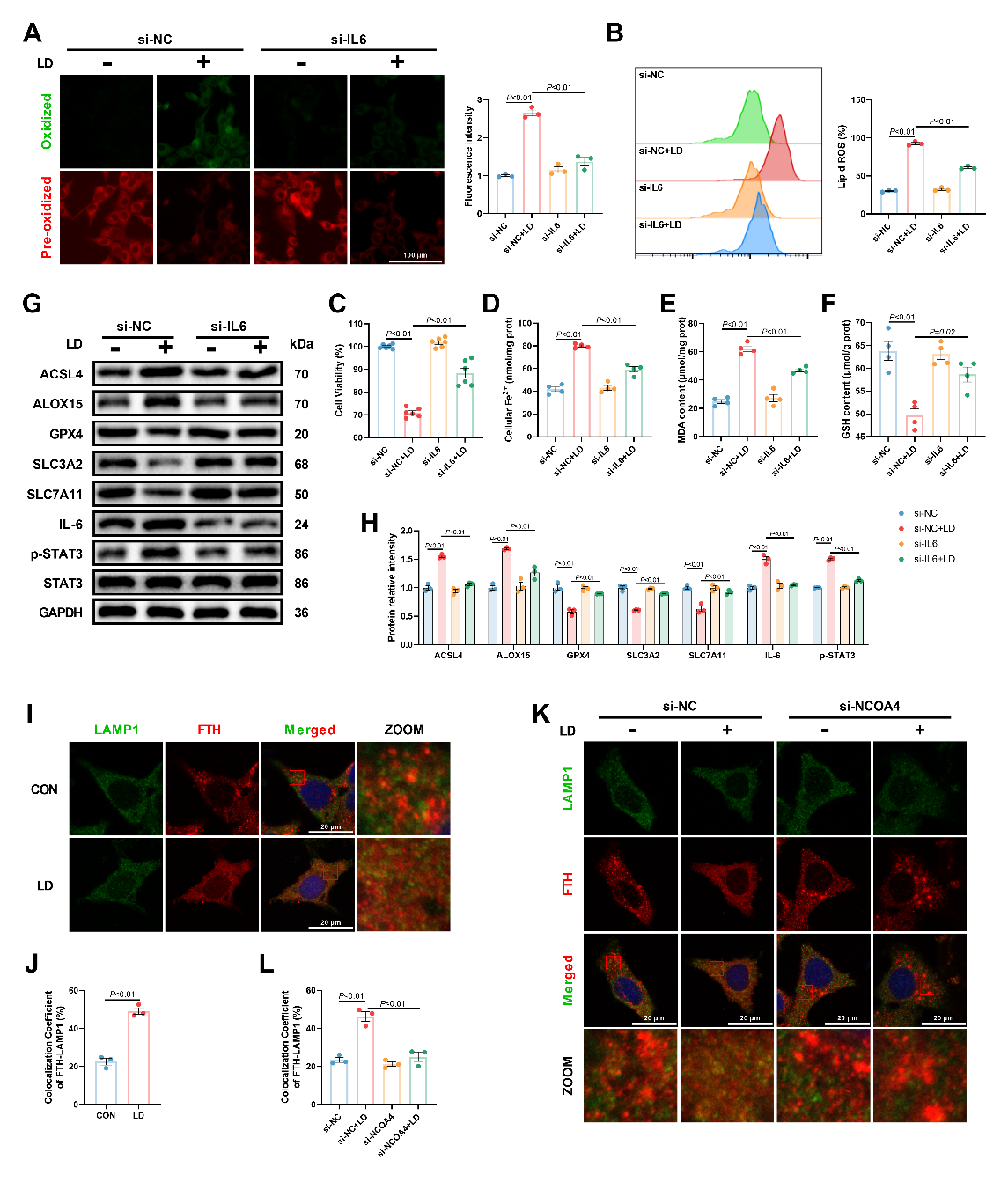


**Fig S6. Inhibition of IL-6/STAT3 signaling counteracted ferroptosis in LPS/iE-DAP-treated hepatocytes.** (A-B) Fluorescence images of lipid ROS observed by confocal microscope (Scale bar = 100 μm) and proportion of lipid ROS positive cells analyzed by flow cytometer. (C) Cell viability, (D) cellular Fe^2+^, (E) MDA content, (F) GSH content, and (G-H) protein expression of ACSL4, ALOX15, GPX4, SLC3A2, SLC7A11, IL-6, p-STAT3, and STAT3 in indicated groups; n = 3-6 per group. (I-L) Colocalization analysis of FTH and LAMP1 in indicated groups; n = 3 per group. Data are presented as mean ± SEM.


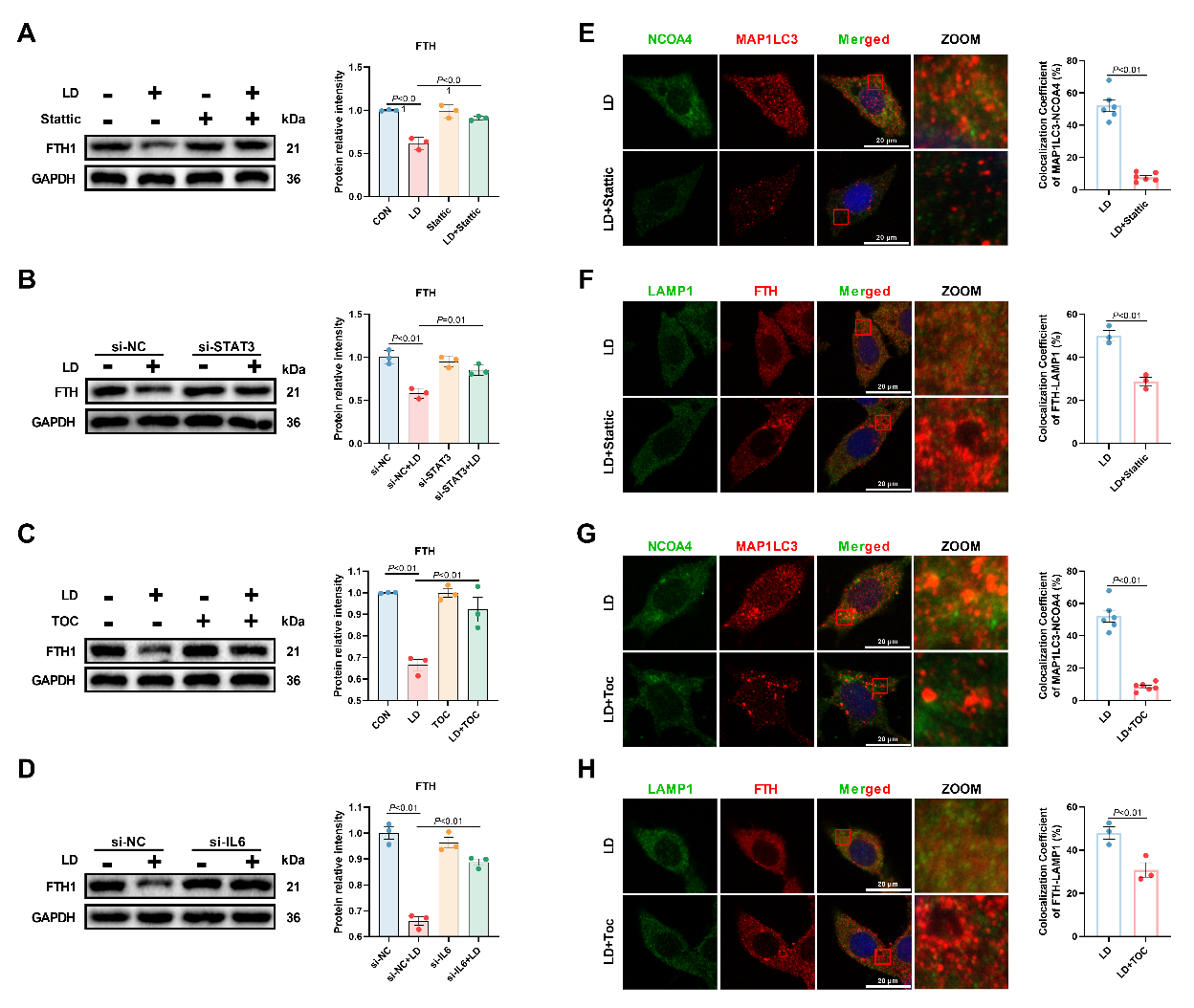


**Fig S7. Inhibition of IL-6/STAT3 signaling led to ferritinophagy blockade.** (A-D) Protein expression of FTH in hepatocytes with different treatments; n = 3 per group. (E-H) Colocalization analysis of MAP1LC3 and NCOA4, and colocalization analysis of FTH and LAMP1 in different groups; n = 3-6 per group. Data are presented as mean ± SEM.


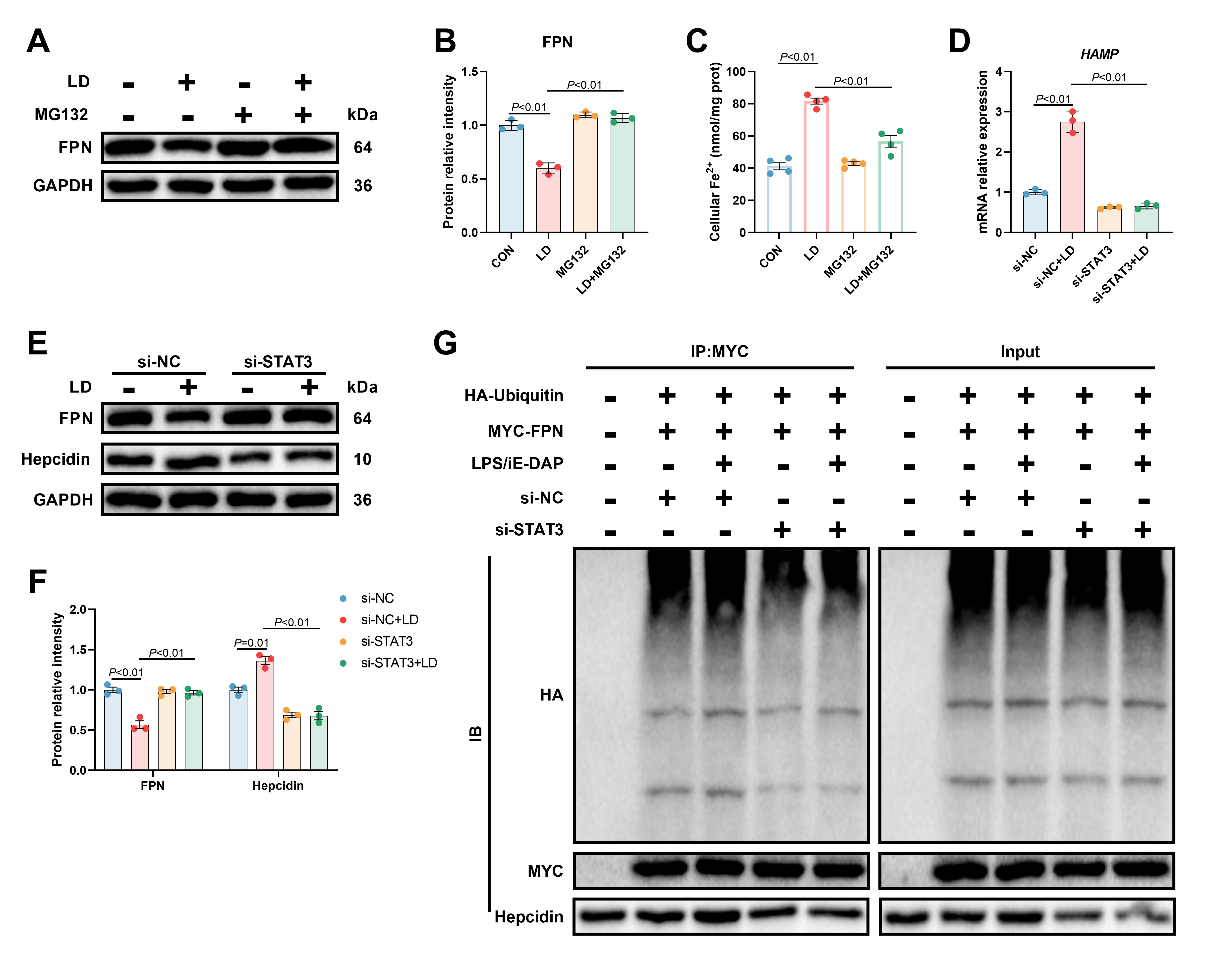


**Fig S8. STAT3 regulated HAMP-dependent FPN degradation.** (A-B) MG132 increased protein expression of FPN and decreased (C) cellular Fe^2+^; n = 3-4 per group (D-F) Gene expression of *HAMP* and protein expression of FPN and hepcidin in indicated groups; n = 3 per group. (G) Coimmunoprecipitation and ubiquitination analysis of FPN in hepatocytes. Data are presented as mean ± SEM.
